# Total Synthesis of the Natural Chalcone Lophirone E, Synthetic Studies toward Benzofuran and Indole-Based Analogues, and Investigation of Anti-Leishmanial Activity

**DOI:** 10.3390/molecules27020463

**Published:** 2022-01-11

**Authors:** Luca Pozzetti, Roberta Ibba, Sara Rossi, Orazio Taglialatela-Scafati, Donatella Taramelli, Nicoletta Basilico, Sarah D’Alessandro, Silvia Parapini, Stefania Butini, Giuseppe Campiani, Sandra Gemma

**Affiliations:** 1 Department of Biotechnology, Chemistry and Pharmacy, University of Siena, Via Aldo Moro 2, 53100 Siena, Italy; luca.pozzetti@student.unisi.it (L.P.); roberta.ibba@unisi.it (R.I.); rossi115@student.unisi.it (S.R.); stefania.butini@unisi.it (S.B.); 2 Department of Pharmacy, School of Medicine and Surgery, University of Naples Federico II, Via D. Montesano 49, 80131 Naples, Italy; scatagli@unina.it; 3 Department of Pharmacological and Biomolecular Sciences, University of Milan, Via Pascal 36, 20133 Milano, Italy; donatella.taramelli@unimi.it (D.T.); sarah.dalessandro@unimi.it (S.D.); 4 Department of Biomedical, Surgical and Dental Sciences, University of Milan, Via Pascal 36, 20133 Milano, Italy; nicoletta.basilico@unimi.it; 5 Department of Biomedical Sciences for Health, University of Milan, Via Pascal 36, 20133 Milano, Italy; silvia.parapini@unimi.it

**Keywords:** chalcones, 2-arylbenzofuran, 2-arylindole, *Leishmania* spp.

## Abstract

The potential of natural and synthetic chalcones as therapeutic leads against different pathological conditions has been investigated for several years, and this class of compounds emerged as a privileged chemotype due to its interesting anti-inflammatory, antimicrobial, antiviral, and anticancer properties. The objective of our study was to contribute to the investigation of this class of natural products as anti-leishmanial agents. We aimed at investigating the structure–activity relationships of the natural chalcone lophirone E, characterized by the presence of benzofuran B-ring, and analogues on anti-leishmania activity. Here we describe an effective synthetic strategy for the preparation of the natural chalcone lophirone E and its application to the synthesis of a small set of chalcones bearing different substitution patterns at both the A and heterocyclic B rings. The resulting compounds were investigated for their activity against *Leishmania infantum* promastigotes disclosing derivatives **1** and **28a**,**b** as those endowed with the most interesting activities (IC_50_ = 15.3, 27.2, 15.9 μM, respectively). The synthetic approaches here described and the early SAR investigations highlighted the potential of this class of compounds as antiparasitic hits, making this study worthy of further investigation.

## 1. Introduction

**Figure 1:**
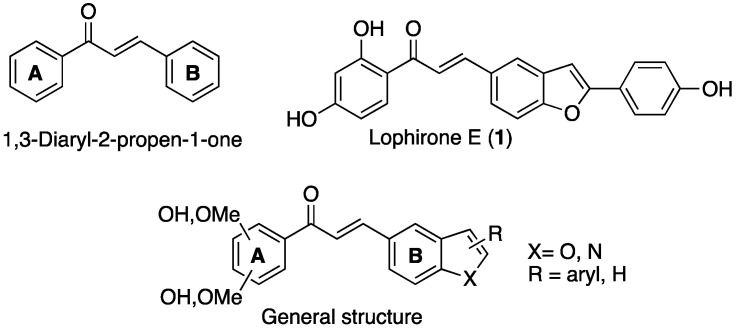
General structures of chalcones and compounds discussed in this work.

Leishmaniasis is a vector-borne parasitic disease transmitted to the human host through the bite of female phlebotomine sandflies. This insect injects the infective, promastigote forms of the parasite, which subsequently are taken up by macrophages and proliferate intracellularly as amastigotes. *Leishmania* species and the phlebotomine sandfly vectors have a widespread distribution in tropical and subtropical countries, including the Mediterranean region. According to WHO data, from 700,000 to 1 million new cases of leishmaniasis occur annually [1]. The most severe form of the disease is the so-called visceral leishmaniasis (VL), mainly due to infections by *L. donovani* and *L. infantum.* VL affects internal organs causing splenomegaly, hepatomegaly, and serious gastrointestinal symptoms. Other forms of leishmaniasis include the cutaneous leishmaniasis (CL), caused by *L. major* and *L. tropica,* which manifests with skin ulcers, and the mucocutaneous leishmaniasis (MCL) due to *L. braziliensis* and characterized by chronic ulcers affecting the mucosal districts. The current therapeutic armamentarium against *Leishmania* lists drugs such as sodium stibogluconate, amphotericin B, eflornithine, paromomycin, and pentamidine, used either alone or in combination [1]. However, these treatments are largely unsatisfactory for several reasons, such as low efficacy in endemic regions, the spread of drug-resistant strains, cost of treatment, safety concerns due to heavy toxicity, length of treatment, and/or suboptimal route of administration. For these reasons, new and more effective therapeutic approaches are urgently required for this debilitating disease. Among the plethora of natural compounds useful for the treatment of infectious diseases, natural chalcones, namely the 1,3-diaryl-2-propen-1-one derivatives (Figure 1), present a relatively simple and yet privileged chemical scaffold [2,3]. Natural chalcones and the related cyclized products, flavanones and flavones, are characterized by a wide range of biological activities and have been exploited in a number of medicinal chemistry applications [4,5,6,7,8,9,10]. Most promising pharmacological activities of chalcones concern their potential utility as anti-diabetic, anti-inflammatory, and anticancer compounds. They have been demonstrated to interfere with key biological pathways involved in the modulation of inflammatory mediators such as COX enzymes, PEG2, NO, or NF-kB. Moreover, selected chalcones are currently under clinical evaluation for the treatment of dermatological conditions [11]. In the field of tropical parasitic diseases, chalcones were reported as a promising new class of antileishmanial agents, showing selective activity in vitro and in vivo against different *Leishmania* species, and characterized by appropriate selectivity indexes [12,13]. The antileishmanial activity of chalcones has been largely investigated through phenotypic assays. The mode of action of synthetic and natural chalcones has not been elucidated yet, even though some targets, such as the arginase enzyme or enzymes involved in the trypanothione metabolic pathways [14,15,16], have been postulated. In addition, licochalcone A has been shown to alter the ultrastructure of the mitochondria and to inhibit the activity of fumarate reductase in the respiratory chain of *L. major* promastigote [17,18]

In this scenario, the aim of our study was to contribute to unveiling the potential of natural and synthetic chalcones as antileishmanial agents through an investigation of heterocyclic ring substitutions on the basic scaffold, which have not yet been investigated in depth. In our continuous effort for the identification of novel compounds as potential anti-leishmania hits [19,20,21,22], we were particularly interested in the evaluation of polyoxygenated natural and synthetic chalcones bearing hydroxyl substituents on the **A** ring and bicyclic heterocycles as the **B** ring (Figure 1). Indeed, the chalcone derivatives possessing hydroxy- and methoxy- substituents on aromatic ring **A** have been reported to have potential anti-leishmanial and anti-trypanosomal activities [14,16]. Moreover, heterocyclic systems in place of the **B** ring have been exploited for the development of antimalarial leads [23,24], but this structural modification has been less investigated for the anti-leishmania activity [12].

Based on this rationale, we took inspiration from the core structure of lophirone E (**1**, Figure 1), characterized by both a polyoxygenated **A** ring and a heterocyclic 2-(4-hydroxyphenyl)benzofuran **B** ring. This is a natural chalcone isolated from the stem barks of *Lophira lanceolata* [25], whose extracts present interesting antiparasitic and anti-inflammatory properties [26,27]. As the first step of our study, we developed a synthetic strategy for the preparation of **1**. We then synthesized a series of benzofuran- and indole-based chalcones (general structure, Figure 1) for early structure–activity relationships (SAR) analysis. All synthesized compounds were tested in a phenotypic assay aimed at elucidating their activity against *L. infantum* promastigotes.

## 2. Results and Discussion

### 2.1. Chemistry

#### 2.1.1. Retrosynthetic Analysis

**Figure 2:**
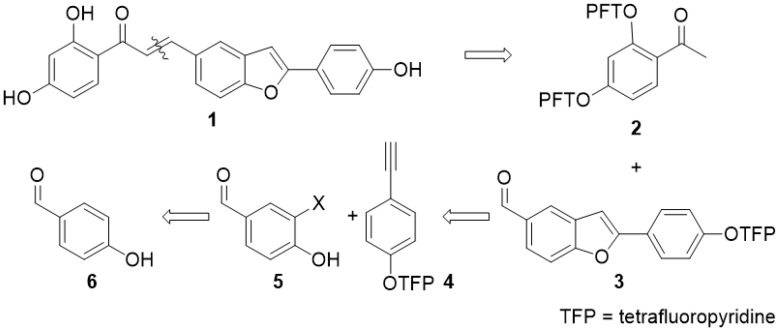
Retrosynthetic analysis for natural chalcone **1**.

The retrosynthetic approach for the preparation of **1** is shown in Figure 2. The first disconnection was performed at the olefin system since it can be obtained by exploiting the Claisen–Schmidt condensation between the appropriately protected 2′,4′-dihydroxyacetophenone and the benzofuran-5-carboxaldehyde derivative **3**. To build the benzofuran core, we chose to apply the Sonogashira cross-coupling reaction by exposing the appropriate *o*-halophenol to a terminal alkyne. Fragment **5** can be obtained via the halogenation of the commercial 4-hydroxybenzaldehyde **6**. Since the presence of several aromatic hydroxyl groups could interfere in both the aldol condensation and the Sonogashira coupling, the choice of a suitable protecting group was of particular importance. The recently proposed tetrafluoropyridyl (TFP) ether has attracted our attention due to its ability to withstand most of the reaction conditions, being sensitive only to strong nucleophiles, along with its ease of installation and cleavage [28].

#### 2.1.2. Synthesis of Synthons **5** and **4**

**Scheme 1:**
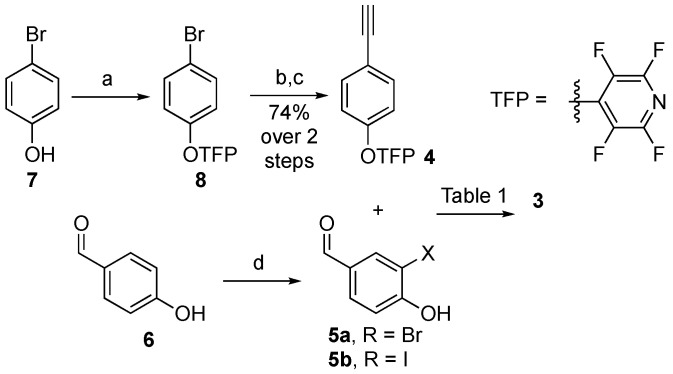
Reagents and Conditions: (**a**) pentafluoropyridine, K_2_CO_3_, ACN, 25 °C, 12 h; (**b**) trimethylsilylacetylene, Pd(PPh_3_)_4_, PPh_3_, CuI, TEA, 90 °C, 3 h; (**c**) TBAF, THF, −78 °C, 5 min; (**d**) *N*-bromosuccinimide, thioacetamide, ACN, 25 °C, 2 h; (**5a**) or AgNO_3_, I_2_, MeOH, 25 °C, 24 h (**5b**).

The key intermediate of the whole retrosynthetic analysis was the benzofuran derivative **3**, whose convergent synthesis is depicted in Scheme 1. 4-Bromophenol **7** was protected by reaction with pentafluoropyridine in the presence of K_2_CO_3_ in acetonitrile, affording compound **8** in quantitative yield. Bromoarene **8** was then submitted to a Sonogashira cross-coupling reaction with trimethylsilylacetylene. This is a well-established procedure that enables the substitution of terminal acetylenic hydrogen with an aryl halide in the presence of a catalytic amount of a palladium catalyst and cuprous iodide in an amine solvent [29]. Deprotection of the silyl-protected terminal alkyne obtained in the previous step was accomplished by treatment with tetrabutylammonium fluoride (TBAF) in anhydrous tetrahydrofuran (THF) at −78 °C for 5 min [30], allowing isolation of the terminal acetylene derivative **4** in 74% yield over two steps. The key step of the synthetic approach for the synthesis of benzofuran aldehyde **3** was a one-pot Sonogashira coupling-intramolecular cyclization between **4** and haloarenes **5a**,**b**, in turn obtained by the halogenation of **6** with NBS for **5a** or with I_2_ in the presence AgNO_3_ for **5b** (Scheme 1 and Table 1). 

#### 2.1.3. Optimization of the One-Pot Sonogashira Coupling-Intramolecular Cyclization to Key Synthon **3**

The first attempts of coupling/cyclization were carried out between TFP-protected 4-ethynylphenol **4** and bromoaldehyde **5a** using Pd(PPh_3_)_4_ as the catalyst, CuI as co-catalyst, and PPh_3_ as ligand in anhydrous triethylamine (TEA) at 90 °C, and did not afford the expected product (Table 1). In fact, after 3 h, no reaction occurred (entry 1). The same protocol, using longer reaction times, resulted in the formation of the terminal alkyne homocoupling product **9** (entry 2) due to the competing Glaser reaction [31]. This side reaction needs an oxidative environment to occur; therefore, it can be avoided by carefully degassing the solvent before adding it to the reaction flask. This expedient reduced the number of homocoupling by-products formed during the reaction, but the yield of the benzofuran still remained low with a variety of catalysts (entries 3 and 4). The best catalytic system appeared to be Pd(PPh_3_)_4_, with increasing yields following the increasing reaction times (entries 5, 6 and 7). To further improve the yield of compound **3**, we used the iodinated derivative **5b** that was more reactive, providing the desired product in 83% yield with no Glaser homocoupling by-product (**9**) (entry 8).

#### 2.1.4. Synthesis of **1**

**Scheme 2:**
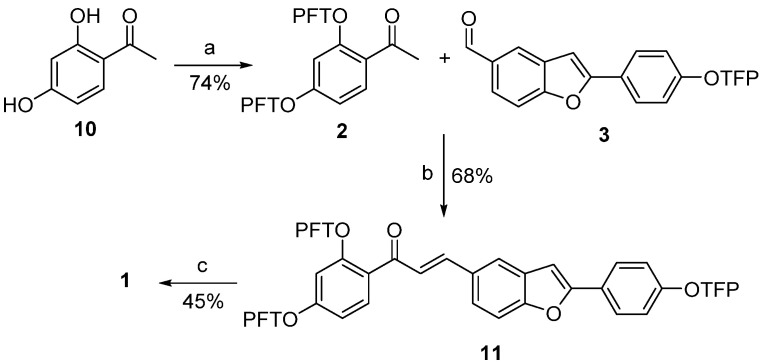
Reagents and Conditions: (**a**) pentafluoropyridine, K_2_CO_3_, ACN, 25 °C, 12 h; (**b**) trimethylsilyl trifluoromethanesulfonate, triethylamine, DCM, −20 °C to 25 °C, 12 h; (**c**) methyl thioglycolate, KF, 18-C-6, ACN/H_2_O, 25 °C, 12 h.

With the assembly of the benzofuran core **3** optimized, we proceeded to the evaluation of the best reaction conditions for the condensation between the aromatic aldehyde **3** and the acetophenone derivative **2**, which was obtained upon the protection of commercially available 2′,4′-dihydroxyacetophenone **10** (Scheme 2). Due to the protection pattern of the building blocks, condensation reactions using strong nucleophiles were not pursued. Since strongly alkaline conditions were revealed to be unsuitable for this reaction, probably due to the low reactivity of the electron-rich heteroaromatic aldehyde towards the enolate of **2**, we turned our attention toward Lewis acid-promoted conditions. Gratifyingly, treatment of a mixture of **2** and **3** with trimethylsilyl trifluoromethanesulfonate (TMSOTf) in the presence of Et_3_N at −20 °C allowed us to obtain the desired compound **11** in 68% yield. The final deprotection step was carried out by careful and slow addition of methyl thioglycolate to a solution of the chalcone, potassium fluoride, and 18-Crown-6. This resulted in the formation of a mixture of partially deprotected products, of the Michael adduct of methyl thioglycolate, and of **1**, which was isolated in 45% yield after RP-18 HPLC purification.

**Figure 3:**
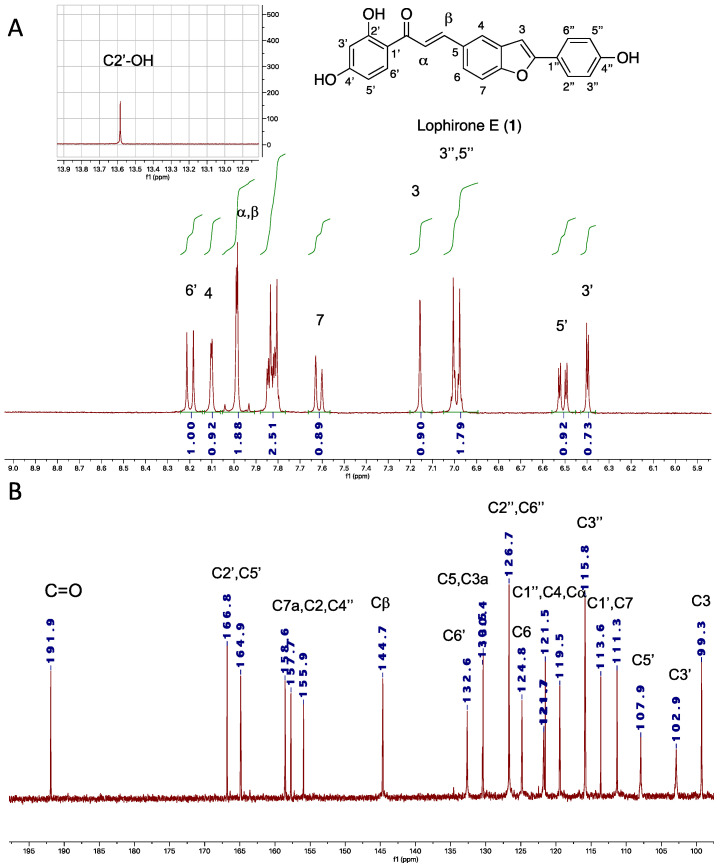
^1^H NMR (**A**) and ^13^C NMR (**B**) of lophirone E (**1**).

The ^1^H and ^13^C NMR spectra of the synthetic lophirone E (**1**) are reported in Figure 3A,B and they are in very good agreement with those reported in the literature [25]. In particular, the 2′-OH is clearly evident at 13.6 ppm, as well as the spin system of the B ring comprising proton signals at 8.19, 6.53 and 6.41 ppm (Figure 3A). Finally, the double bond proton signals at 8.01 and 7.96 ppm present *J* values (15.5 Hz) typical of the trans geometry. ^13^C carbons (Figure 3B) have been assigned based on the spectral analysis reported in [25].

#### 2.1.5. Optimization of the Synthetic Approach and Synthesis of Chalcone Derivatives **16**–**18** and **21a,b**

For the synthesis of the planned analogues, to be used for early structure-activity relationships, we implemented a different synthetic approach foreseeing the use of a MOM-protecting group instead of TFP, which in the previous synthetic approach induced the observed constraints in the purification of the final compound. 

**Scheme 3:**
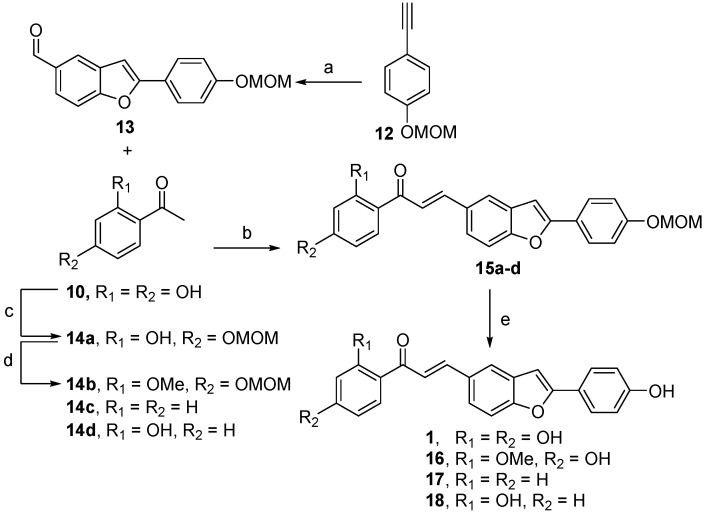
Reagents and Conditions: (**a**) **5b**, Pd(PPh_3_)_4_, PPh_3_, CuI, TEA, 90 °C, 12 h; (**b**) NaOH (5 M), EtOH, 0 °C to 25 °C, 72 h; (**c**) MOMCl, DIPEA, dry DCM, 25 °C, 2 h; (**d**) K_2_CO_3_, MeI, DMF, 25 °C, 12 h; (**e**) HCl (12 M), MeOH/THF, 0 °C to 40 °C, 1 h.

As described in Scheme 3, acetylene **12** was prepared as described in the literature [32], and **5b** were submitted to the previously described one-pot Sonogashira coupling-intramolecular cyclization conditions to furnish the MOM-protected benzofuranaldehyde **13**. Aldehyde **13** was then reacted with the appropriate MOM-protected acetophenones **14a**–**d** to afford the corresponding condensation products **15a**–**d**. While ketones **14c**,**d** were commercially available, intermediate **14a** was prepared from **10** by treatment with methoxymethyl- (MOM-) chloride, while **14b** was obtained from **14a** upon a methylation reaction. 

**Scheme 4:**
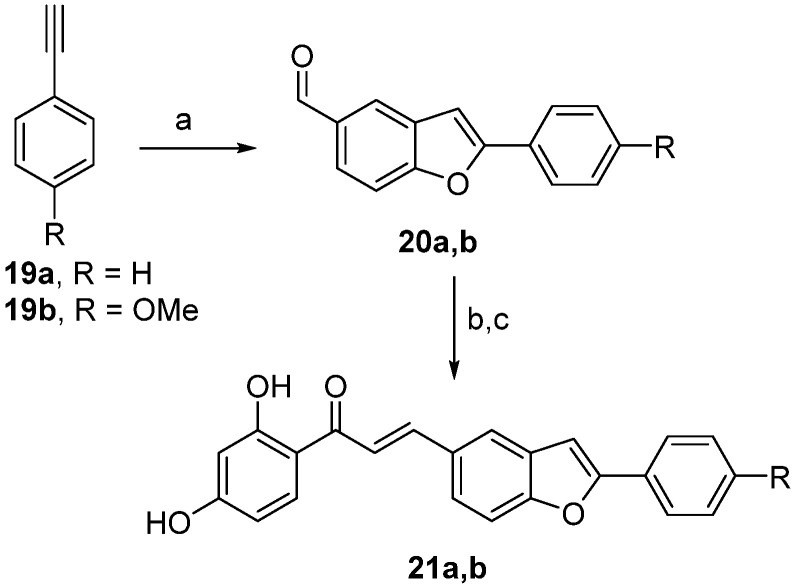
Reagents and Conditions: (**a**) **5b**, Pd(PPh_3_)_4_, PPh_3_, CuI, TEA, 90 °C, 12 h; (**b**) **14a**, NaOH (5 M), EtOH, 0 °C to 25 °C, 72 h; (**c**) HCl (12 M), MeOH/THF, 0 °C to 40 °C, 1 h.

It is worth noting that for the preparation of **15a** and **15d**, no protection of the free OH at the C2′ position was necessary, probably because the 2′-hydroxyl substituent is involved in an intramolecular H-bond with the carbonyl oxygen, that greatly reduces its reactivity. The final deprotection of the MOM ethers of **15a**–**d** with HCl_conc_ in warm MeOH/THF gave **1** and its analogues **16**–**18** in good to excellent yields. Regarding the preparation of **1**, the slightly lower yield of the condensation step with respect to the previous approach was balanced by the cleaner deprotection step, which afforded the natural compound in 69% yield. Further analogues, **21a**,**b**, were prepared as described in Scheme 4. Accordingly, acetylenes **19a**,**b** were converted into the corresponding benzofuran aldehydes **20a**,**b** by condensation with **5b** as described above. The condensation of these latter aldehydes with acetophenone **14a**, followed by acid-catalyzed deprotection of the MOM ether, afforded the final chalcones in reasonable yields.

#### 2.1.6. Synthesis of Indole Derivatives **25a**,**b** and **28a**,**b**

**Scheme 5:**
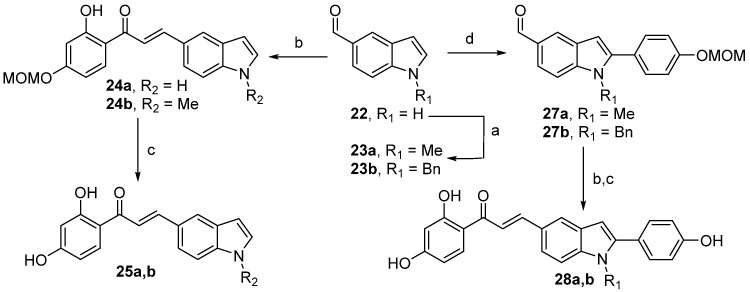
Reagents and Conditions: (**a**) NaH (60% *w*/*w*), CH_3_I (for **23a**) or BnBr (for **23b**), DMF, 0 °C to 25 °C, 12 h; (**b**) **14a**, NaOH (5 M), EtOH, 0 °C to 25 °C, 48 h; (**c**) HCl (12 M), MeOH/THF, 0 °C to 40 °C, 1 h; (**d**) **26**, Ag_2_O, 2-nitrobenzoic acid, Pd(OAc)_2_, DMF, 25 °C, 24 h.

Indole-based chalcones **25a**,**b** and **28a**,**b** were prepared as described in Scheme 5. Commercially available indole-5-carbaldehyde **22** was either *N*-methylated or *N*-benzylated to give compounds **23a** and **23b**, respectively. Aldehydes **22** and **23a** were condensed with **14a** to afford **24a**,**b** and these latter intermediates were finally deprotected to the desired compounds **25a**,**b**. The pendant phenol substituent at C2 of the indole ring was placed by a reaction of **23a**,**b** with 4-iodo-1-(methoxymethyl)phenate [32] in the presence of Ag_2_O, 2-nitrobenzoic acid and Pd(OAc)_2_ to give the corresponding 2-aryl-substituted indole-5-carbaldehydes **27a**,**b**. These latter intermediates were submitted to the aldol condensation with acetophenone derivative **14a** to give, after HCl-mediated deprotection, chalcones **28a**,**b**.

### 2.2. Evaluation of Chalcone Derivatives against Leishmania Promastigotes

As reported in Table 2, chalcones **1**, **16**–**18**, **21a**,**b**, **25a**,**b**, and **28a**,**b** were tested in vitro against promastigotes of *L. infantum*, responsible for visceral leishmaniasis in the Mediterranean basin.

Compound **1** displayed an IC_50_ value of 15.3 μM against *Leishmania* promastigotes, thus becoming four times more potent than the reference chalcone **29** bearing an unsubstituted phenyl **B** ring and prepared by a novel synthetic strategy (see Scheme S1 in the Supporting Material). The first set of analogues **16**–**18** was prepared to assess the role of the 2,4-dihydroxy substituents on activity. From the evaluation of these analogues, it can be deduced that a free OH at C2′ is necessary for activity since both **16** and **17** displayed IC_50_ values higher than 65 μM. Alongside derivatives **21a**,**b**, we also investigated the role of the 4-hydroxy substituent at the pendant 2-arylbenzofuran. Removal of the 4″-OH group is tolerated since **21a** has an IC_50_ value comparable to derivative **1**, but its replacement with a more sterically hindered MeO- group resulted in a drop of potency. When the 2-aryl-benzofuran moiety was replaced by an indole (**25a**) or an *N*-Me indole (**25b**), a reduction and a loss of potency were observed, respectively. The anti-leishmania potency was recovered when a 2-aryl substituent was placed at C2 of the indole moiety (**28a**,**b**). The activity of **28b** is also interesting since it presents a pendant benzyl group that could be used for future exploitation.

## 3. Materials and Methods

### 3.1. General

All chemicals, reagents, solvents and chromatography stationary phases were purchased from commercial sources (Merck KGaA, Darmstadt, Germany) and used without any further purification unless otherwise specified. Reaction progress was monitored by thin-layer chromatography (TLC, ), carried out on silica (60 F254) or alumina (60 F254, basic) gel plates with detection by UV, and the products were purified using either silica (60 M, 0.040–0.063 μm) or alumina (90, standardized) column chromatography. ^1^H and ^13^C NMR spectra were recorded in the indicated deuterated solvent on a 300 MHz spectrometer (Varian, Inc. Palo Alto, CA, USA) using the residual signal of the deuterated solvent as an internal standard. Splitting patterns are indicated by s (singlet), d (doublet), dd (doublet of doublets), t (triplet), q (quartet), m (multiplet), and br (broad); chemical shifts (*δ*) are described in parts per million (ppm) and coupling constants (*J*) in Hertz (Hz). ESI-MS spectra were performed by an Agilent 1100 Series LC/MSD spectrometer (Agilent Technologies, Inc. Santa Clara, CA, USA). Yields refer to purified products and are not optimized. All moisture-sensitive reactions were performed under a nitrogen atmosphere using oven-dried glassware and freshly distilled anhydrous solvents. Semi-preparative HPLC was performed on a Shimadzu Prominence apparatus (Shimadzu Corporation, Kyoto, Japan) equipped with a scanning absorbance UV-VIS detector (Diode Array SPD-M20A) also equipped with a thermostatic chamber and Purosphere^®^ STAR RP-18 endcapped (5 μm), 250–10 HPLC column.

### 3.2. Experimental Procedures

*General Procedure (**A**) for the Sonogashira coupling-intracellular cyclization to benzofuran intermediates*. To a mixture of the appropriate *o*-halophenol (1 eq., 1 mmol), Pd(PPh_3_)_4_ (0.05 eq., 0.05 mmol), PPh_3_ (0.055 eq., 0.055 mmol), and CuI (0.05 eq., 0.05 mmol) under an N_2_ atmosphere, a degassed solution of the appropriate terminal alkyne (3 eq., 3 mmol) in anhydrous Et_3_N (6 mL) was added dropwise. The mixture was heated to 90 °C and stirred for 12 h. The resulting dark suspension was acidified with HCl (1 M) and extracted with EtOAc (3 × 5 mL). The combined organic phases were washed with NaHCO_3_ (s.s., 1 × 5 mL) and brine (1 × 5 mL), dried over Na_2_SO_4_, and evaporated under reduced pressure. The residue was purified by silica gel column chromatography.

*General procedure (**B**) for the aldol condensation between substituted acetophenones and substituted aromatic aldehydes*. To a solution of the appropriate acetophenone (1 eq., 1 mmol) in absolute EtOH (3 mL), cooled to 0 °C, NaOH_aq._ (5 M, 5 mmol) was added dropwise. The mixture was stirred at 0 °C for 30 min, and then a solution of the appropriate aromatic aldehyde (1.2 eq., 1.2 mmol) in absolute EtOH (1.5 mL) was added dropwise. The ice bath was removed, and the mixture was stirred at 25 °C for 48 h. The reaction was acidified with HCl (1 M) and extracted with EtOAc (3 × 5 mL). The combined organic phases were washed with NaHCO_3_ (s.s., 1 × 10 mL) and brine (1 × 5 mL), dried over Na_2_SO_4_, and evaporated under reduced pressure. The residue was purified by silica gel column chromatography.

*General Procedure (**C**) for the methoxymethyl ether cleavage.* To a 0 °C cooled solution of the appropriate chalcone (1 eq., 1 mmol) in MeOH/THF (1:1, 20 mL in total), HCl (12 M, 20 eq., 20 mmol) was added dropwise. The ice bath was removed, and the reaction mixture was heated to 40 °C for 3 h. After cooling, NaHCO_3_ (s.s.) was carefully added until pH = 7, and the aqueous phase was extracted with EtOAc (3 × 10 mL). The combined organic phases were washed with brine (1 × 15 mL), dried over Na_2_SO_4_, and evaporated under reduced pressure. The residue was purified by silica gel column chromatography.

*3-Bromo-4-hydroxybenzaldehyde (***5a***)*. To a stirred solution 4-hydroxybenzaldehyde (**6**, 1 eq., 1 mmol) in ACN (5 mL), a solution of *N*-bromosuccinimide (1.1 eq., 1.1 mmol) and thioacetamide (0.05 eq., 0.05 mmol) in ACN (5 mL) was added dropwise over 2 h using a dropping funnel. The mixture was stirred for 2 h, then quenched with Na_2_S_2_O_3_ (s.s., 10 mL) and extracted with EtOAc. (3 × 5 mL). The combined organic phases were washed with brine (5 mL), dried over Na_2_SO_4_, and evaporated under reduced pressure. The crude residue was purified by silica gel column chromatography (DCM/Hex 9:1) to afford the title compound (37% yield) as a white solid. ^1^H NMR (300 MHz, CDCl_3_) *δ* 9.83 (s, 1H, C*H*O), 8.04 (d, *J* = 1.9 Hz, 1H, C6-*H*), 7.77 (dd, *J* = 8.4, 1.9 Hz, 1H, C5-*H*), 7.15 (d, *J* = 8.4 Hz, 1H, C2-*H*), 6.30 (s, 1H, O*H*). ESI-MS, *m*/*z* [*M*-H]^−^ 200 and 202 (1:1 ratio).

*3-Iodo-4-hydroxybenzaldehyde (***5b***)*. To a stirred solution of 4-hydroxybenzaldehyde (**6**, 1 eq., 1 mmol) and AgNO_3_ (1 eq., 1 mmol) in MeOH (6 mL) at 25 °C, I_2_ (1.1 eq., 1.1 mmol) was added. The mixture was stirred for 24 h, quenched with Na_2_S_2_O_3_ (s.s., 10 mL), and extracted with EtOAc. (3 × 5 mL). The combined organic phases were washed with brine (5 mL), dried over Na_2_SO_4_, and evaporated under reduced pressure. The crude residue was purified by silica gel column chromatography (DCM/Hex 15:1) to afford the title compound (44% yield) as an off-white solid. ^1^H NMR (300 MHz, CDCl_3_) δ 9.83 (s, 1H C*H*O), 8.04 (d, *J* = 1.9 Hz, 1H. C6-*H*), 7.77 (dd, *J* = 8.4, 1.9 Hz, 1H, C5-*H*), 7.15 (d, *J* = 8.4 Hz, 1H, C2-*H*), 6.30 (s, 1H, O*H*). ESI-MS *m*/*z* 247 [*M*-H]^−^.

*4-(4-Bromophenoxy)-2,3,5,6-tetrafluoropyridine (***8***)*. To a stirred suspension of 4-bromophenol (**7**, 1 eq., 1 mmol) and anhydrous K_2_CO_3_ (1.05 eq., 1.05 mmol) in can (5 mL) at 25 °C, pentafluoropyridine (1.05 eq., 1.05 mmol) was added. The reaction mixture was stirred for 12 h, quenched with HCl (1 M, 10 mL), and extracted with EtOAc (3 × 5 mL). The combined organic phases were washed with brine (5 mL), dried over Na_2_SO_4_, and evaporated under reduced pressure to afford the title compound (quantitative yield) as a colourless oil without further purification. ^1^H NMR (300 MHz, CDCl_3_) *δ* 7.50 (d, *J* = 9.1 Hz, 2H, C2-*H* and C6-*H*), 6.96 (d, *J* = 9.1 Hz, 2H, C3-*H* and C5-*H*). ^19^F NMR (282 MHz, CDCl_3_) *δ* −88.21 (m), −151.66–−157.75 (m). ESI-MS, *m*/*z* [*M+*H]^+^ 322 and 324 (1:1 ratio).

*4-(4-Ethynylphenoxy)-2,3,5,6-tetrafluoropyridine (***4***).* To a mixture of 4-(4-bromophenoxy)-2,3,5,6-tetrafluoropyridine (**8**, 1eq., 1 mmol), Pd(PPh_3_)_4_ (0.05 eq., 0.05 mmol), PPh_3_ (0.055 eq., 0.05 mmol), and CuI (0.05 eq., 0.05 mmol) under N_2_ atmosphere, a degassed solution of trimethylsilylacetylene (1.5 eq., 1.5 mmol) in anhydrous Et_3_N (6 mL) was added dropwise at 25 °C. The mixture was heated to 90 °C and stirred for 4 h. The dark suspension was acidified with HCl (1 M) and extracted with EtOAc (3 × 5 mL). The combined organic phases were washed with NaHCO_3_ (s.s., 5 mL) and brine (5 mL), dried over Na_2_SO_4_, and evaporated under reduced pressure. The crude residue was purified by silica gel column chromatography (Pet. Et./EtOAc 10:1) to afford 2,3,5,6-tetrafluoro-4-(4-((trimethylsilyl)ethynyl)phenoxy)pyridine (93% yield) as a transparent oil. ^1^H NMR (300 MHz, CDCl_3_) *δ* 7.48 (d, *J* = 8.9 Hz, 2H, C2-*H* and C6-*H*), 6.98 (d, *J* = 8.7 Hz, 2H, C3-*H* and C5-*H*), 0.25 (s, 9H, Si*Me_3_*). ^19^F NMR (282 MHz, CDCl_3_) *δ* −88.18–−88.41 (m), −153.93–−154.16 (m). ESI-MS, *m*/*z* [*M+*H]^+^ 340. To solution of the above-mentioned compound (1 eq., 1 mmol) in anhydrous THF (5 mL), cooled to −78 °C, TBAF (1.0 M solution in THF, 1 eq., 1 mmol) was added dropwise. After 5 min, the reaction was quenched with NaHCO_3_ (s.s., 10 mL) and extracted with EtOAc (3 × 5 mL). The combined organic phases were washed with brine (5 mL), dried over Na_2_SO_4_, and evaporated under reduced pressure. The crude residue was purified by silica gel column chromatography (Pet. Et./EtOAc 100:1) to afford the title compound (80% yield) as a yellow oil. ^1^H NMR (300 MHz, CDCl_3_) *δ* 7.50 (d, *J* = 8.9 Hz, 2H, C2-*H* and C6-*H*), 7.01 (d, *J* = 8.9 Hz, 2H, C3-*H* and C5-*H*), 3.08 (s, 1H). ^19^F NMR (282 MHz, CDCl_3_) *δ* −88.24 (m), −149.41–−162.97 (m). ESI-MS, *m*/*z* [*M+*H]^+^ 268.

*2-(4-((Perfluoropyridin-4-yl)oxy)phenyl)benzofuran-5-carbaldehyde (***3***)*. Following the General Procedure **A**, starting from 3-iodo-4-hydroxybenzaldehyde (**5b**) and 4-(4-ethynylphenoxy)-2,3,5,6-tetrafluoropyridine (**4**), the title compound was obtained as a yellow solid (83% yield) after purification by silica gel column chromatography (Hex/Acetone 6:1). ^1^H NMR (300 MHz, CDCl_3_) *δ* 10.07 (s, 1H, C*H*O), 8.14 (d, *J* = 1.6 Hz, 1H, C4-*H*), 7.88 (m, 3H, C6-*H* and C2′-*H*, C6′-*H*), 7.64 (d, *J* = 8.5 Hz, 1H, C7-*H*), 7.17 (d, *J* = 9.0 Hz, 2H, C3′-*H*, C5′-*H*), 7.11 (s, 1H, C3-*H*). ^19^F NMR (282 MHz, CDCl_3_) *δ* −88.04 (m), −148.90–−162.97 (m). ESI-MS, *m*/*z* [*M*+H]^+^ 388.

*1-(2,4-bis((Perfluoropyridin-4-yl)oxy)phenyl)ethanone (***2***).* To a stirred suspension of 1-(2,4-dihydroxyphenyl)ethanone (**10**, 1 eq., 1 mmol) and anhydrous K_2_CO_3_ (2.5 eq., 2.5 mmol) in ACN (5 mL), pentafluoropyridine (2.5 eq., 2.5 mmol) was added. The reaction mixture was stirred at 25 °C for 12 h, quenched with HCl (1 M, 10 mL), and the aqueous phase was extracted with EtOAc (3 × 5 mL). The combined organic phases were washed with brine (5 mL), dried over Na_2_SO_4_, and evaporated under reduced pressure. The crude residue was purified by silica gel column chromatography (Pet.Et./DCM 2:1) to afford the title compound (74% yield) as a white solid. ^1^H NMR (300 MHz, CDCl_3_) *δ* 7.95 (d, *J* = 8.7 Hz, 1H, C6-*H*), 6.89 (dd, *J* = 8.7, 2.3 Hz, 1H, C5-*H*), 6.77 (d, *J* = 2.3 Hz, 1H, C3-*H*), 2.64 (s, 3H, C*H*_3_). ^19^F NMR (282 MHz, CDCl_3_) *δ* −85.04–−90.34 (m), −150.28–−158.26 (m). ESI-MS, *m*/*z* [*M+*H]^+^ 451.

*(E)-1-(2,4-bis((Perfluoropyridin-4-yl)oxy)phenyl)-3-(2-(4-((perfluoropyridin-4-yl)oxy)phenyl)benzofuran-5-yl)prop-2-en-1-one (***11***)*. To a solution of 1-(2,4-bis((perfluoropyridin-4-yl)oxy)phenyl)ethanone (**2**, 1 eq., 1 mmol), 2-(4-((perfluoropyridin-4-yl)oxy)phenyl)benzofuran-5-carbaldehyde (**3**, 1.2 eq., 1.2 mmol), and anhydrous Et_3_N (1 eq., 1 mmol) in anhydrous DCM (5 mL), cooled to −20 °C, TMSOTf (1 eq., 1 mmol) was added dropwise. The cooling bath was removed, and the reaction was stirred at 25 °C for 2 h. The addition of TMSOTf (1 eq., 1 mmol) was repeated two more times at −20 °C every 2 h. The reaction was quenched with NaHCO_3_ (s.s., 10 mL) and extracted with DCM (3 × 5 mL). The combined organic phases were washed with brine (5 mL), dried over Na_2_SO_4_, and evaporated under reduced pressure. The crude residue was purified by silica gel column chromatography (Hex/Acetone 6:1) to afford the title compound (68% yield) as a yellow oil. ^1^H NMR (300 MHz, Acetone-*d*_6_) *δ* 8.07–8.00 (m, 3H, Ar-*H*), 7.98 (d, *J* = 8.6 Hz, 1H, Ar-*H*), 7.84–7.73 (m, 2H, Ar-*H* and (*E*)-CαH=Cβ*H*), 7.64 (d, *J* = 8.6 Hz, 1H, Ar-*H*), 7.49 (d, *J* = 16.0 Hz, 1H, (*E*)-Cα*H*=CβH), 7.44 (m, 3H, Ar-*H*), 7.40–7.33 (m, 2H, Ar-*H*). ^19^F NMR (282 MHz, Acetone-*d*_6_) *δ* −88.09–−96.64 (m), −152.75–−165.21 (m). ESI-MS, *m*/*z* [*M+*H]^+^ 820.

*(E)-1-(2,4-Dihydroxyphenyl)-3-(2-(4-hydroxyphenyl)benzofuran-5-yl)prop-2-en-1-one (***1***)*. To a suspension of (*E*)-1-(2,4-bis((perfluoropyridin-4-yl)oxy)phenyl)-3-(2-(4-((perfluoropyridin-4-yl)oxy)phenyl)benzofuran-5-yl)prop-2-en-1-one (**11**, 1 eq., 1 mmol), KF (3 eq., 3 mmol), 18-C-6 (3 eq., 3 mmol) in ACN/H_2_O (1 mL/20 μL), cooled to 0 °C, a solution of methyl thioglycolate (2 eq., 2 mmol) in ACN/H_2_O (1 mL/20 μL) was added dropwise. The cooling bath was removed, and the mixture was stirred for 1 h. The addition of methyl thioglycolate (2 eq., 2 mmol) in ACN/H_2_O (1 mL/20 μL) was repeated two more times at 1 h intervals. The solvent was removed under reduced pressure, the crude residue was suspended in H_2_O (2 mL), and the aqueous phase was extracted with EtOAc (3 × 2 mL). The combined organic phases were washed with brine (2 × 2 mL), dried over Na_2_SO_4_, and evaporated under reduced pressure. The crude residue was purified by semi-preparative HPLC (Purosphere^®^ STAR RP-18 endcapped 5 μm, 250-10), eluting with ACN/H_2_O (0.1% TFA *v*/*v*) (1:10 → 8:10 over 30 min, 8:10 for 5 min, 8:10 → 9.5:10 over 5 min) to afford the title compound (45% yield) as an orange solid. Spectroscopic data are identical to those reported for the natural compound. ^1^H NMR (300 MHz, Acetone-*d*_6_) *δ* 13.59 (s, 1H, C2′O*H*), 8.19 (d, *J* = 8.9 Hz, 1H, C6′-*H*), 8.10 (d, *J* = 1.6 Hz, 1H, C4-*H*), 8.01 (d, *J* = 15.5 Hz, 1H, (*E*)-CαH=Cβ*H*), 7.96 (d, *J* = 15.5 Hz, 1H, (*E*)-Cα*H*=CβH), 7.89–7.78 (m, 3H, C6-*H*, C2″-*H* and C6″-*H*), 7.62 (d, *J* = 8.5 Hz, 1H, C7-*H*), 7.16 (d, *J* = 0.8 Hz, 1H, C3-*H*), 7.00 (d, *J* = 8.8 Hz, 2H, C3″-*H* and C5″-*H*), 6.53 (dd, *J* = 8.9, 2.4 Hz, 1H, C5′-*H*), 6.41 (d, *J* = 2.4 Hz, 1H, C3′-*H*). ^13^C NMR (75 MHz, Acetone-*d*_6_) *δ* 191.89, 166.78, 164.86, 158.54, 157.70, 155.92, 144.64, 132.62, 130.43, 130.35, 126.66 (2C), 124.82, 121.70, 121.52, 119.44, 115.83 (2C), 113.62, 111.29, 107.91, 102.85, 99.24. ESI-MS, *m*/*z* [*M*-H]^−^ 371.

*2-(4-(Methoxymethoxy)phenyl)benzofuran-5-carbaldehyde (***13***)*. Following the General Procedure **A**, starting from 3-iodo-4-hydroxybenzaldehyde (**5b**) and 1-ethynyl-4-(methoxymethoxy)benzene (**12**), the title compound was obtained as a yellow oil (89% yield) after purification by silica gel column chromatography (Pet. Et./EtOAc 8:1). ^1^H NMR (300 MHz, CDCl_3_) *δ* 10.05 (s, 1H, C*H*O), 8.08 (s, 1H, C4-*H*), 7.86–7.68 (m, 3H, C3′-*H*, C5′-*H* and C6-*H*), 7.60 (d, *J* = 8.5 Hz, 1H, C7-*H*), 7.13 (d, *J* = 8.7 Hz, 2H, C2′-*H*, C6′-*H*), 6.98 (s, 1H, C3-*H*), 5.24 (s, 2H, OC*H*_2_OCH_3_), 3.51 (s, 3H, OCH_2_OC*H*_3_). ESI-MS, *m*/*z* [*M+*H]^+^ 283.

*1-(2-Hydroxy-4-(methoxymethoxy)phenyl)ethan-1-one (***14a***)*. To a solution of 1-(2,4-dihydroxyphenyl)ethan-1-one (**10**, 1 eq., 1 mmol) in anhydrous DCM (10 mL), DIPEA (1.5 eq., 1.5 mmol) was added, followed by chloromethyl methyl ether (1.5 eq., 1.5 mmol). The mixture was stirred at 25 °C for 2 h. The reaction mixture was washed with HCl (1 M) (2 × 5 mL), with NaHCO_3_ (s.s., 5 mL), and brine (5 mL). The organic phase was dried over Na_2_SO_4_ and evaporated under reduced pressure to obtain the title compound with no further purification (quantitative yield) as a yellow oil. ^1^H NMR (300 MHz, CDCl_3_) *δ* 12.60 (s, 1H, C2′O*H*), 7.65 (d, *J* = 8.8 Hz, 1H, C6′-*H*), 6.59 (d, *J* = 2.4 Hz, 1H, C3′-*H*), 6.55 (dd, *J* = 8.8, 2.4 Hz, 1H, C5′-*H*), 5.20 (s, 2H, OC*H*_2_OCH_3_), 3.47 (s, 3H, OCH_2_OC*H*_3_), 2.56 (s, 3H, COC*H*_3_). ESI-MS, *m*/*z* [*M*-H]^−^ 195.

*1-(2-Methoxy-4-(methoxymethoxy)phenyl)ethan-1-one (***14b***)*. To a solution of 1-(2-hydroxy-4-(methoxymethoxy)phenyl)ethan-1-one (**14a**, 1 eq., 1 mmol) in anhydrous DMF (6 mL) under N_2_ atmosphere, K_2_CO_3_ (1.5 eq., 1.5 mmol) was added, followed by CH_3_I (1.5 eq., 1.5 mmol). The mixture was stirred at 25 °C for 12 h. Then the reaction was quenched with NH_4_Cl (s.s., 5 mL) and extracted with EtOAc (3 × 5 mL). The combined organic phases were washed with NaHCO_3_ (s.s., 5 mL) and brine (5 mL), dried over Na_2_SO_4_, and evaporated under reduced pressure. The crude residue was purified by silica gel column chromatography (Pet. Et./EtOAc 4:1) to afford the title compound (37% yield) as a transparent oil. ^1^H NMR (300 MHz, CDCl_3_) *δ* 7.77 (d, *J* = 8.7 Hz, 1H, C6′-*H*), 6.62 (dd, *J* = 8.7, 2.2 Hz, 1H, C5′-*H*), 6.58 (d, *J* = 2.1 Hz, 1H, C3′-*H*), 5.18 (s, 2H, OC*H*_2_OCH_3_), 3.86 (s, 3H, C2′OC*H*_3_), 3.45 (s, 3H, OCH_2_OC*H*_3_), 2.54 (s, 3H, COC*H*_3_). ESI-MS, *m*/*z* [*M* + Na]^+^ 233.

*2-Phenylbenzofuran-5-carbaldehyde (***20a***)*. Following the General Procedure **A**, starting from 3-iodo-4-hydroxybenzaldehyde (**5b**) and ethynylbenzene (**19a**), the title compound was obtained as a yellow solid (65% yield) after purification by silica gel column chromatography (Pet. Et./EtOAc 20:1). ^1^H NMR (300 MHz, CDCl_3_) *δ* 10.05 (s, 1H, C*H*O), 8.10 (s, 1H, C4-*H*), 7.86 (m, 3H, C6-*H*, C2-*ArH*), 7.62 (d, *J* = 8.5 Hz, 1H, C7-*H*), 7.54–7.36 (m, 3H, C2-*ArH*), 7.09 (s, 1H, C3-*H*). ESI-MS, *m*/*z* [*M+*H]^+^ 223.

*2-(4-Methoxyphenyl)benzofuran-5-carbaldehyde (***20b***)*. Following the General Procedure **A**, starting from 3-iodo-4-hydroxybenzaldehyde (**5b**) and 1-ethynyl-4-methoxybenzene (**19b**), the title compound was obtained as an off-white solid (67% yield) after purification by silica gel column chromatography (Pet. Et./EtOAc 7:1). ^1^H NMR (300 MHz, CDCl_3_) *δ* 10.05 (s, 1H, C*H*O), 8.08 (s, 1H, C4-*H*), 7.92–7.68 (m, 3H, C6-*H*, C3′-*H* and C5′-*H*), 7.59 (d, *J* = 8.5 Hz, 1H, C7-*H*), 6.99 (d, *J* = 8.8 Hz, 2H, C2′-*H* and C6′-*H*), 6.96 (s, 1H, C3-*H*), 3.87 (s, 3H, ArOC*H_3_*). ESI-MS, *m*/*z* [*M+*H]^+^ 253.

*(E)-1-(2-Hydroxy-4-(methoxymethoxy)phenyl)-3-(2-(4-(methoxymethoxy)phenyl)benzofuran-5-yl)prop-2-en-1-one (***15a***)*. Following the General Procedure **B**, starting from 1-(2-hydroxy-4-(methoxymethoxy)phenyl)ethan-1-one (**14a**) and 2-(4-(methoxymethoxy)phenyl)benzofuran-5-carbaldehyde (**13**), the title compound was obtained as an orange solid (56% yield) after purification by silica gel column chromatography (Pet. Et./EtOAc 4:1). ^1^H NMR (300 MHz, CDCl_3_) *δ* 13.39 (s, 1H, C2′O*H*), 8.00 (d, *J* = 15.4 Hz, 1H, CαH=Cβ*H*), 7.89 (d, *J* = 9.0 Hz, 1H, C6′-*H*), 7.86–7.74 (m, 3H, Ar-*H* and Cα*H*=CβH), 7.69–7.49 (m, 3H, Ar-*H*), 7.13 (d, *J* = 8.8 Hz, 2H, C3″-*H* and C5″-*H*), 6.92 (s, 1H, C3-*H*), 6.66 (d, *J* = 2.4 Hz, 1H, C3′-*H*), 6.60 (dd, *J* = 8.8, 2.4 Hz, 1H, C5′-*H*), 5.23 (s, 4H), 3.51 (s, 6H). ESI-MS, *m*/*z* [*M*-H]^−^ 459.

*(E)-1-(2-Methoxy-4-(methoxymethoxy)phenyl)-3-(2-(4-(methoxymethoxy)phenyl)benzofuran-5-yl)prop-2-en-1-one (***15b***)*. Following the General Procedure **B**, starting from 1-(2-methoxy-4-(methoxymethoxy)phenyl)ethan-1-one (**14b**) and 2-(4-(methoxymethoxy)phenyl)benzofuran-5-carbaldehyde (**13**), the title compound was obtained as a yellow solid (63% yield) after purification by silica gel column chromatography (Pet. Et./EtOAc 2:1). ^1^H NMR (300 MHz, CDCl_3_) *δ* 7.76 (m, 5H Ar-*H* and CαH=Cβ*H*), 7.50 (m, 3H, Ar-*H*, and Cα*H*=CβH), 7.10 (d, *J* = 8.6 Hz, 2H, C3″-*H*, C5″-*H*), 6.88 (s, 1H, C3-*H*), 6.71 (d, *J* = 8.6 Hz, 1H, C5′-*H*), 6.65 (s, 1H, C3′-*H*), 5.22 (s, 2H, OC*H_2_*OCH_3_), 5.21 (s, 2H, OC*H_2_*OCH_3_), 3.91 (s, 3H, C2′OC*H_3_*), 3.50 (s, 3H, OCH_2_OC*H*_3_), 3.49 (s, 3H, OCH_2_OC*H*_3_). ESI-MS, *m*/*z* [*M*+Na]^+^ 497.

*(E)-3-(2-(4-(Methoxymethoxy)phenyl)benzofuran-5-yl)-1-phenylprop-2-en-1-one (***15c***)*. Following the General Procedure **B**, starting from acetophenone (**14c**) and 2-(4-(methoxymethoxy)phenyl)benzofuran-5-carbaldehyde (**13**), the title compound was obtained as an orange solid (48% yield) after purification by silica gel column chromatography (Pet. Et./EtOAc 4:1).^1^H NMR (300 MHz, CDCl_3_) *δ* 8.05 (d, *J* = 7.0 Hz, 2H, C2′-*H* and C6′-*H*), 7.94 (d, *J* = 15.7 Hz, 1H, CαH=Cβ*H*), 7.83 (s, 1H, Ar-*H*), 7.80 (d, *J* = 8.8 Hz, 2H, C2″-*H*, C6″-*H*), 7.64–7.56 (m, 4H, Ar-*H* and Cα*H*=CβH), 7.53 (d, *J* = 7.0 Hz, 2H, C3′-*H* and C5′-*H*), 7.13 (d, *J* = 8.7 Hz, 2H, C3″-*H*, C5″-*H*), 6.93 (s, 1H, C3-*H*), 5.24 (s, 2H, OC*H_2_*OCH_3_), 3.51 (s, 3H, OCH_2_OC*H*_3_). ESI-MS, *m*/*z* [*M*+H]^+^ 385.

*(E)-1-(2-Hydroxyphenyl)-3-(2-(4-(methoxymethoxy)phenyl)benzofuran-5-yl)prop-2-en-1-one (***15d***)*. Following the General Procedure **B**, starting from 1-(2-hydroxyphenyl)ethan-1-one (**14d**) and 2-(4-(methoxymethoxy)phenyl)benzofuran-5-carbaldehyde (**13**), the title compound was obtained as an orange solid (47% yield) after purification by silica gel column chromatography (Pet. Et./EtOAc 4:1). ^1^H NMR (300 MHz, CDCl_3_) *δ* 12.95 (s, 1H, C2′O*H*), 8.00 (m, 1H, Ar-*H*), 7.84 (s, 1H, Ar-*H*), 7.80 (d, *J* = 8.2 Hz, 2H, C2″-*H*, C6″-*H*), 7.68 (m, 1H, Ar-*H*), 7.62 (m, 1H, Ar-*H*), 7.57–7.40 (m, 3H, Ar-*H*), 7.13 (d, *J* = 8.1 Hz, 2H, C3″-*H*, C5″-*H*), 7.04 (m, 1H, Ar-*H*), 6.99 (m, 1H, Ar-*H*), 6.92 (s, 1H, C3-*H*), 5.23 (s, 2H, OC*H_2_*OCH_3_), 3.51 (s, 3H, OCH_2_OC*H*_3_). ESI-MS, *m*/*z* [*M*-H]^−^ 399.

*1-Methyl-1H-indole-5-carbaldehyde (***23a***)*. To a solution of 1*H*-indole-5-carbaldehyde (**22**, 1 eq., 1 mmol) in anhydrous DMF (5 mL), cooled to 0 °C NaH (60% *w*/*w*, 1.5 eq., 1.5 mmol) was added under N_2_ atmosphere. The suspension was stirred at the same temperature for 30 min, then CH_3_I (1.5 eq., 1.5 mmol) was added dropwise. The ice bath was removed, and the reaction mixture was stirred at 25 °C for 12 h. The reaction was quenched with NH_4_Cl (s.s., 5 mL) and extracted with EtOAc (3 × 5 mL). The combined organic phases were washed with NaHCO_3_ (s.s., 5 mL) and brine (5 mL), dried over Na_2_SO_4_, and evaporated under reduced pressure. The crude residue was purified by silica gel column chromatography (Pet. Et./EtOAc 4:1) to afford the title compound (91% yield) as a white solid. ^1^H NMR (300 MHz, CDCl_3_) *δ* 10.03 (s, 1H, C*H*O), 8.15 (s, 1H, C4-*H*), 7.80 (d, *J* = 8.9 Hz, 1H, C6-*H*), 7.40 (d, *J* = 8.6 Hz, 1H, C7-*H*), 7.15 (d, *J* = 3.2 Hz, 1H, C2-*H*), 6.65 (d, *J* = 3.1 Hz, 1H, C3-*H*), 3.85 (s, 3H, NC*H_3_*). ESI-MS, *m*/*z* [*M*+H]^+^ 160.

*1-Benzyl-1H-indole-5-carbaldehyde (***23b***)*. Starting from 1*H*-indole-5-carbaldehyde **22**, the title compounds were obtained as a yellow oil in 84% yield after purification by silica gel column chromatography (Pet. Et./EtOAc 8:1) following the procedure described for the preparation of **23a**. ^1^H NMR (300 MHz, CDCl_3_) *δ* 10.03 (s, 1H, C*H*O), 8.18 (s, 1H, C4-*H*), 7.75 (d, *J* = 8.6 Hz, 1H, C6-*H*), 7.37 (d, *J* = 8.6 Hz, 1H, C7-*H*), 7.32 (m, 3H, NCH_2_*Ph*), 7.23 (d, *J* = 3.2 Hz, 1H, C3-*H*), 7.11 (m, 2H, NCH_2_*Ph*), 6.72 (d, *J* = 3.2 Hz, 1H, C2-*H*), 5.37 (s, 2H, NC*H_2_*Ph). ESI-MS, *m*/*z* [*M*+H]^+^ 236.

*2-(4-(Methoxymethoxy)phenyl)-1-methyl-1H-indole-5-carbaldehyde (***27a***)*. A suspension of Pd(OAc)_2_, 1-iodo-4-(methoxymethoxy)benzene (**26**), 2-nitrobenzoic acid, Ag_2_O, and 1-methyl-1*H*-indole-5-carbaldehyde (**23a**) in anhydrous DMF under N_2_ atmosphere was stirred at 25 °C for 24 h. Then, the reaction mixture was filtered through Celite^®^ using EtOAc as eluent and washed with NH_4_Cl (s.s., 5 mL), NaHCO_3_ (s.s., 5 mL), and brine (5 mL). The organic phase was dried over Na_2_SO_4_ and evaporated under reduced pressure. The crude residue was purified by silica gel column chromatography (Pet. Et./EtOAc 2:1) to afford the title compound (71% yield) as a yellow oil. ^1^H NMR (300 MHz, CDCl_3_) *δ* 10.03 (s, 1H, C*H*O), 8.13 (s, 1H, C4-*H*), 7.79 (d, *J* = 8.6 Hz, 1H, C6-*H*), 7.42 (m, 3H, C7-*H*, C3′-*H* and C5′-*H*), 7.16 (d, *J* = 8.7 Hz, 2H, C2′-*H* and C6′-*H*), 6.64 (s, 1H, C3-*H*), 5.25 (s, 2H, OC*H_2_*OCH_3_), 3.75 (s, 3H, NC*H_3_*), 3.53 (s, 3H, OCH_2_OC*H_3_*). ESI-MS, *m*/*z* [*M*+H]^+^ 296.

*1-Benzyl-2-(4-(methoxymethoxy)phenyl)-1H-indole-5-carbaldehyde (***27b***)*. Starting from 1-iodo-4-(methoxymethoxy)benzene (**26**) and -benzyl-1*H*-indole-5-carbaldehyde (**23b**), the title compounds were obtained as a yellow oil in 80% yield after purification by silica gel column chromatography (Pet. Et./EtOAc 5:1) following the procedure described for the preparation of **27a**. ^1^H NMR (300 MHz, CDCl_3_) *δ* 9.97 (s, 1H, C*H*O), 8.13 (s, 1H, C4-*H*), 7.66 (d, *J* = 8.5 Hz, 1H, C6-*H*), 7.32 (d, *J* = 8.7 Hz, 2H, C3′-*H* and C5′-*H*), 7.21 (m, 4H, C7-*H* and NCH_2_*Ph*), 7.04 (d, *J* = 8.7 Hz, 2H, C2′-*H* and C6′-*H*), 6.95 (m, 2H, NCH_2_*Ph*), 6.69 (s, 1H, C3-*H*), 5.34 (s, 2H, NC*H_2_*Ph), 5.16 (s, 2H, OC*H_2_*OCH_3_), 3.45 (s, 3H, OCH_2_OC*H_3_*). ESI-MS, *m*/*z* [*M*+H]^+^ 372.

*(E)-1-(2-Hydroxy-4-(methoxymethoxy)phenyl)-3-(1H-indol-5-yl)prop-2-en-1-one (***24a***)*. Following the General Procedure **B**, starting from 1-(2-hydroxy-4-(methoxymethoxy)phenyl)ethan-1-one (**14a**) and 1*H*-indole-5-carbaldehyde (**22**), the title compound was obtained as a yellow solid (53% yield) after purification by silica gel column chromatography (Pet. Et./EtOAc 4:1). ^1^H NMR (300 MHz, CDCl_3_) *δ* 13.52 (s, 1H, C2′O*H*), 8.44 (br, 1H, N*H*), 8.07 (d, *J* = 15.3 Hz, 1H, CαH=Cβ*H*), 7.95 (s, 1H, C4-*H*), 7.90 (d, *J* = 9.0 Hz, 1H, C6′-*H*), 7.62–7.52 (m, 2H, Cα*H*=CβH and C6-*H*), 7.43 (d, *J* = 8.5 Hz, 1H, C7-*H*), 7.27 (m, 1H, C2-*H*), 6.68–6.54 (m, 3H, Ar′-*H* and C3-*H*), 5.23 (s, 2H, OC*H_2_*OCH_3_), 3.49 (s, 3H, OCH_2_OC*H_3_*). ESI-MS, *m*/*z* [*M*-H]^−^ 322.

*(E)-1-(2-Hydroxy-4-(methoxymethoxy)phenyl)-3-(1-methyl-1H-indol-5-yl)prop-2-en-1-one (***24b***)*. Following the General Procedure **B**, starting from 1-(2-hydroxy-4-(methoxymethoxy)phenyl)ethan-1-one (**14a**) and 1-methyl-1*H*-indole-5-carbaldehyde (**23a**), the title compound was obtained as a yellow oil (60% yield) after purification by silica gel column chromatography (Pet. Et./EtOAc 4:1). ^1^H NMR (300 MHz, CDCl_3_) *δ* 13.53 (s, 1H, C2′O*H*), 8.08 (d, *J* = 15.3 Hz, 1H, CαH=Cβ*H*), 7.98–7.86 (m, 2H, C4-*H* and C6′-*H*), 7.58 (d, *J* = 15.4 Hz, 1H, Cα*H*=CβH), 7.58 (m, 1H, C6-*H*), 7.36 (d, *J* = 8.6 Hz, 1H, C7-*H*), 7.10 (d, *J* = 3.1 Hz, 1H, C2-*H*), 6.65 (d, *J* = 2.4 Hz, 1H, C3′-*H*), 6.60 (dd, *J* = 8.9, 2.4 Hz, 1H, C5′-*H*), 6.56 (d, *J* = 3.1 Hz, 1H, C3-*H*), 5.23 (s, 2H, OC*H_2_*OCH_3_), 3.83 (s, 3H, NC*H_3_*), 3.50 (s, 3H, OCH_2_OC*H_3_*). ESI-MS, *m*/*z* [*M*-H]^−^ 336.

*(E)-1-(2,4-Dihydroxyphenyl)-3-(2-(4-hydroxyphenyl)benzofuran-5-yl)prop-2-en-1-one (***1***).* Following the General Procedure **C**, starting from *(E)*-1-(2-hydroxy-4-(methoxymethoxy)phenyl)-3-(2-(4-(methoxymethoxy)phenyl)benzofuran-5-yl)prop-2-en-1-one (**15a**), the title compound was obtained as an orange solid (69% yield) after purification by silica gel column chromatography (DCM/MeOH 20:1). Spectroscopic data (^1^H NMR, ^13^C NMR, ESI-MS) were in agreement with the previously reported for the same compound.

*(E)-1-(4-Hydroxy-2-methoxyphenyl)-3-(2-(4-hydroxyphenyl)benzofuran-5-yl)prop-2-en-1-one (***16***)*. Following the General Procedure **C**, starting from (*E*)-1-(2-methoxy-4-(methoxymethoxy)phenyl)-3-(2-(4-(methoxymethoxy)phenyl)benzofuran-5-yl)prop-2-en-1-one (**15b**) the title compound was obtained as an orange solid (69% yield) after purification by silica gel column chromatography (DCM/MeOH 100:1). ^1^H NMR (300 MHz, Acetone-*d*_6_) *δ* 7.93 (s, 1H, C4-*H*), 7.81 (d, *J* = 8.7 Hz, 2H, C2″-*H* and C6″-*H*), 7.73 (d, *J* = 15.7 Hz, 1H, CαH=Cβ*H*), 7.69–7.61 (m, 3H, Cα*H*=CβH, C6-*H* and C7-*H*), 7.57 (d, *J* = 8.6 Hz, 1H, C6′-*H*), 7.13 (s, 1H, C3-*H*), 6.99 (d, *J* = 8.7 Hz, 2H, C3″-*H* and C5″-*H*), 6.61 (d, *J* = 2.0 Hz, 1H, C3′-*H*), 6.55 (dd, *J* = 8.5, 2.1 Hz, 1H, C5′-*H*), 3.95 (s, 3H). ^13^C NMR (75 MHz, Acetone-*d*_6_) *δ* 188.83, 162.79, 160.97, 158.56, 157.50, 155.52, 141.29, 132.66, 130.91, 130.37, 126.60 (2C), 126.34, 124.10, 121.53, 121.03, 120.95, 115.84 (2C), 111.22, 107.86, 99.27, 99.15, 55.23. ESI-MS, *m*/*z* [*M*-H]^−^ 385.

*(E)-3-(2-(4-Hydroxyphenyl)benzofuran-5-yl)-1-phenylprop-2-en-1-one (***17***)*. Following the General Procedure **C**, starting from (*E*)-3-(2-(4-(methoxymethoxy)phenyl)benzofuran-5-yl)-1-phenylprop-2-en-1-one (**15c**) the title compound was obtained as an orange solid (81% yield) after purification by silica gel column chromatography (DCM/MeOH 100:1). ^1^H NMR (300 MHz, Acetone-*d*_6_) *δ* 8.87 (br, 1H, C4″O*H*), 8.18 (m, 2H, Ar-*H*), 8.08 (s, 1H, Ar-*H*), 7.94 (d, *J* = 15.7 Hz, 1H, CαH=Cβ*H*), 7.88 (d, *J* = 15.7 Hz, 1H, Cα*H*=CβH), 7.87–7.77 (m, 3H, C2″-*H*, C6″-*H* and Ar-*H*), 7.67–7.51 (m, 4H, Ar-*H*), 7.15 (s, 1H, C3-*H*), 6.99 (d, *J* = 8.7 Hz, 2H, C3″-*H*, C5″-*H*). ESI-MS, *m*/*z* [*M*-H]^−^ 339.

*(E)-1-(2-Hydroxyphenyl)-3-(2-(4-hydroxyphenyl)benzofuran-5-yl)prop-2-en-1-one (***18***)*. Following the General Procedure **C**, starting from (*E*)-1-(2-hydroxyphenyl)-3-(2-(4-(methoxymethoxy)phenyl)benzofuran-5-yl)prop-2-en-1-one (**15d**) the title compound was obtained as an orange solid (74% yield) after purification by silica gel column chromatography (DCM/MeOH 100:1). ^1^H NMR (300 MHz, Acetone-*d*_6_) *δ* 13.02 (s, 1H, C2′O*H*), 8.33 (d, *J* = 8.0 Hz, 1H, C6′-*H*), 8.14 (s, 1H, C4-*H*), 8.08 (s, 2H, Cα*H*=Cβ*H*), 7.87 (dd, *J* = 8.6, 1.6 Hz, 1H, C6-*H*), 7.82 (d, *J* = 8.7 Hz, 2H, C2″-*H* and C6″-*H*), 7.67–7.53 (m, 2H, C7-H and C4′-*H*), 7.16 (s, C3-*H*), 7.05–6.93 (m, 2H, C3′-*H* and C5′-*H*), 7.00 (d, *J* = 8.7 Hz, 2H, C3″-*H* and C5″-*H*). ^13^C NMR (75 MHz, Acetone-*d*_6_) *δ* 193.97, 163.63, 163.29, 158.56, 157.82, 156.12, 146.09, 136.38, 130.49, 130.14, 126.67 (2C), 125.06, 122.03, 121.42, 120.03, 119.20, 118.94, 117.92, 115.79 (2C), 111.37, 99.22. ESI-MS, *m*/*z* [*M*-H]^−^ 355.

*(E)-1-(2,4-Dihydroxyphenyl)-3-(2-phenylbenzofuran-5-yl)prop-2-en-1-one (***21a***)*. Following the General Procedure **B**, starting from 1-(2-hydroxy-4-(methoxymethoxy)phenyl)ethan-1-one (**14a**) and 2-phenylbenzofuran-5-carbaldehyde (**20a**), (*E*)-1-(2-hydroxy-4-(methoxymethoxy)phenyl)-3-(2-phenylbenzofuran-5-yl)prop-2-en-1-one was obtained as a yellow solid (58% yield) after purification by silica gel column chromatography (Pet. Et./EtOAc 4:1). ^1^H NMR (300 MHz, CDCl_3_) *δ* 13.58 (s, 1H, C2′O*H*), *δ* 7.86 (m, 2H, Ar-*H*), 7.82–7.73 (m, 2H, Ar-*H* and CαH=Cβ*H*), 7.70 (d, *J* = 8.6 Hz, 1H, C6′-*H*), 7.59–7.42 (m, 5H, Ar-*H* and Cα*H*=CβH), 7.38 (m, 1H, Ar-*H*), 7.02 (s, 1H, C3-*H*), 6.88 (d, *J* = 2.2 Hz, 1H, C3′-*H*), 6.79 (dd, *J* = 8.6, 2.2 Hz, 1H, C5′-*H*), 5.27 (s, 2H), 5.23 (s, 2H, OC*H_2_*OCH_3_), 3.51 (s, 3H, OCH_2_OC*H*_3_). ESI-MS, *m*/*z* [*M*-H]^−^ 399. The abovementioned compound was then submitted to the General Procedure **C**, thus obtaining the title compound as a yellow solid (93% yield) after purification by silica gel column chromatography (Pet. Et./EtOAc 1:1). ^1^H NMR (300 MHz, Acetone-*d*_6_) *δ* 13.58 (s, 1H, C2′O*H*), 9.57 (br, 1H, C4′O*H*), 8.21 (d, *J* = 8.9 Hz, 1H, C6′-*H*), 8.16 (s, 1H, C4-*H*), 7.99 (m, 4H, Cα*H*=Cβ*H*, Ar-*H*), 7.90 (d, *J* = 8.6, 1H, C6-*H*), 7.67 (d, *J* = 8.5 Hz, 1H, C7-*H*), 7.53 (m, 2H, Ar-*H*), 7.48–7.35 (m, 2H, Ar-*H*), 6.50 (dd, *J* = 8.8, 2.4 Hz, 1H, C5′-*H*), 6.39 (d, *J* = 2.3 Hz, 1H, C3′-*H*). ^13^C NMR (75 MHz, Acetone-*d*_6_) *δ* 191.88, 166.79, 164.88, 157.06, 156.13, 144.45, 132.66, 130.56, 130.04, 129.90, 129.07, 128.98 (2C), 125.44, 124.89 (2C), 122.29, 119.69, 113.61, 111.58, 107.91, 102.84, 101.66. ESI-MS, *m*/*z* [*M*-H]^−^ 355. 

*(E)-1-(2-Hydroxy-4-(methoxymethoxy)phenyl)-3-(2-(4-methoxyphenyl)benzofuran-5-yl)prop-2-en-1-one (***21b***)*. Following the General Procedure **B**, starting from 1-(2-hydroxy-4-(methoxymethoxy)phenyl)ethan-1-one (**14a**) and 2-(4-methoxyphenyl)benzofuran-5-carbaldehyde (**20b**), (*E*)-1-(2-hydroxy-4-(methoxymethoxy)phenyl)-3-(2-(4-methoxyphenyl)benzofuran-5-yl)prop-2-en-1-one was obtained as a yellow solid (44% yield) after purification by silica gel column chromatography (Pet. Et./EtOAc 2:1). ^1^H NMR (300 MHz, CDCl_3_) *δ* 13.57 (s, 1H, C2′O*H*), *δ* 7.78 (m, 4H, Ar-*H*), 7.69 (d, *J* = 8.6 Hz, 1H, C6′-*H*), 7.49 (m, 3H, Ar-*H*), 6.98 (m, 2H, Ar-*H*), 6.88 (m, 2H, Ar-*H*), 6.79 (d, *J* = 8.5 Hz, 1H, C5′-*H*), 5.27 (s, 2H, OC*H_2_*OCH_3_), 3.85 (s, 3H, C4″OC*H_3_*), 3.51 (s, 3H, OCH_2_OC*H*_3_). ESI-MS, *m*/*z* [*M*-H]^−^ 429. The abovementioned compound was then submitted to the General Procedure **C**, thus obtaining the title compound as a yellow solid (91% yield) after purification by silica gel column chromatography (Pet. Et./EtOAc 1:1). ^1^H NMR (300 MHz, *d*-DMSO) *δ* 13.54 (s, 1H, C2′O*H*), 8.23 (d, *J* = 8.9 Hz, 1H), 8.16 (s, 1H, C4-*H*), 7.99 (d, *J* = 15.4 Hz, 1H, CαH=Cβ*H*), 7.92 (d, *J* = 15.3 Hz, 1H, Cα*H*=CβH), 7.88 (d, *J* = 8.7 Hz, 2H, C2″-*H* and C6″-*H*), 7.85 (m, 1H, C6-*H*), 7.67 (d, *J* = 8.6 Hz, 1H, C7-*H*), 7.33 (s, 1H, C3-*H*), 7.07 (d, *J* = 8.7 Hz, 2H, C3″-*H* and C5″-*H*), 6.42 (dd, *J* = 8.9, 1.7 Hz, 1H, C5′-*H*), 6.28 (d, *J* = 1.7 Hz, 1H, C3′-*H*), 3.81 (s, 3H, C4″OC*H_3_*). ^13^C NMR (75 MHz, *d*-DMSO) *δ* 191.54, 166.62, 166.43, 160.49, 157.09, 155.82, 144.51, 133.48, 130.60, 130.25, 126.92 (2C), 125.80, 122.45, 120.55, 115.02 (2C), 113.10, 112.29, 111.91, 109.12, 103.13, 100.67, 55.75. ESI-MS, *m*/*z* [*M*-H]^−^ 385.

*(E)-1-(2,4-Dihydroxyphenyl)-3-(1H-indol-5-yl)prop-2-en-1-one (***25a**, CAS registry number 2412944-18-2*)*. Following the General Procedure **C**, starting from (*E*)-1-(2-hydroxy-4-(methoxymethoxy)phenyl)-3-(1*H*-indol-5-yl)prop-2-en-1-one (**24a**) the title compound was obtained as a yellow solid (83% yield) after purification by silica gel column chromatography (DCM/MeOH 100:1). ^1^H NMR (300 MHz, CD_3_OD) *δ* 7.98 (m, 3H, CαH=Cβ*H*, C6′-*H* and C4-*H*), 7.72 (d, *J* = 15.3 Hz, 1H, CαH=Cβ*H*), 7.60 (d, *J* = 8.6 Hz, 1H, C6-*H*), 7.43 (d, *J* = 8.5 Hz, 1H, C7-*H*), 7.28 (d, *J* = 2.9 Hz, 1H, C2-*H*), 6.53 (d, *J* = 3.0 Hz, 1H, C3-*H*), 6.43 (dd, *J* = 8.6, 1.8 Hz, 1H, C5′-*H*), 6.30 (d, *J* = 2.0 Hz, 1H, C3′-*H*). ^13^C NMR (75 MHz, CD_3_OD) *δ* 192.19, 166.09, 164.87, 146.64, 137.91, 131.93, 128.43, 126.16, 125.59, 122.89, 121.07, 116.42, 113.32, 111.46, 107.66, 102.34, 102.07. ESI-MS, *m*/*z* [*M*-H]^−^ 278.

*(E)-1-(2,4-Dihydroxyphenyl)-3-(1-methyl-1H-indol-5-yl)prop-2-en-1-one (***25b***)*. Following the General Procedure **C**, starting from (*E*)-1-(2-hydroxy-4-(methoxymethoxy)phenyl)-3-(1-methyl-1H-indol-5-yl)prop-2-en-1-one (**24b**) the title compound was obtained as a yellow solid (77% yield) after purification by silica gel column chromatography (DCM/MeOH 100:1). ^1^H NMR (300 MHz, Acetone-*d*_6_) *δ* 13.74 (s, 1H, C2′O*H*), 9.48 (s, 1H, C4′O*H*), 8.19 (d, *J* = 8.9 Hz, 1H, C6′-*H*), 8.07 (s, 1H, C4-*H*), 8.05 (d, *J* = 15.3 Hz, 1H, CαH=Cβ*H*), 7.89 (d, *J* = 15.3 Hz, 1H, Cα*H*=CβH), 7.76 (d, *J* = 8.6 Hz, 1H, C6-*H*), 7.49 (d, *J* = 8.6 Hz, 1H, C7-*H*), 7.31 (d, *J* = 3.1 Hz, 1H, C2-*H*), 6.54 (d, *J* = 3.1 Hz, 1H, C3-*H*), 6.49 (dd, *J* = 8.8, 2.2 Hz, 1H, C5′-*H*), 6.38 (d, *J* = 2.2 Hz, 1H, C3′-*H*), 3.88 (s, 3H, NC*H_3_*). ^13^C NMR (75 MHz, Acetone-*d*_6_) *δ* 191.98, 166.71, 164.55, 146.44, 138.31, 132.42, 130.63, 128.97, 126.30, 123.39, 121.63, 117.04, 113.68, 110.07, 107.72, 102.82, 101.78, 32.21. ESI-MS, *m*/*z* [*M*-H]^−^ 292.

*(E)-1-(2,4-Dihydroxyphenyl)-3-(2-(4-hydroxyphenyl)-1-methyl-1H-indol-5-yl)prop-2-en-1-one (***28a***)*. Following the General Procedure **B**, starting from 1-(2-hydroxy-4-(methoxymethoxy)phenyl)ethan-1-one (**14a**) and 2-(4-(methoxymethoxy)phenyl)-1-methyl-1H-indole-5-carbaldehyde (**27a**), (*E*)-1-(2-hydroxy-4-(methoxymethoxy)phenyl)-3-(2-(4-(methoxymethoxy)phenyl)-1-methyl-1H-indol-5-yl)prop-2-en-1-one was obtained as a yellow oil (67% yield) after purification by silica gel column chromatography (Pet. Et./EtOAc 2:1). ^1^H NMR (300 MHz, CDCl_3_) *δ* 13.47 (s, 1H, C2′O*H*), *δ* 7.83 (s, 1H, C4-*H*), 7.82 (d, *J* = 15.6 Hz, 1H, CαH=Cβ*H*), 7.66 (d, *J* = 8.6 Hz, 1H, C6′-*H*), 7.53 (d, *J* = 8.6 Hz, 1H, C6-*H*), 7.43 (d, *J* = 15.7 Hz, 1H, Cα*H*=CβH), 7.43 (d, *J* = 8.7 Hz, 2H, C2″-*H* and C6″-*H*), 7.34 (d, *J* = 8.6 Hz, 1H, C7-*H*), 7.15 (d, *J* = 8.7 Hz, 2H, C3″-*H* and C5″-*H*), 6.87 (d, *J* = 2.1 Hz, 1H, C3′-*H*), 6.78 (dd, *J* = 8.6, 2.2 Hz, 1H, C5′-*H*), 6.53 (s, 1H, C3-*H*), 5.23 (s, 4H, 2xOC*H_2_*OCH_3_), 3.74 (s, 3H, NC*H_3_*), 3.51 (s, 6H, 2xOCH_2_OC*H_3_*). ESI-MS, *m*/*z* [*M*-H]^−^ 472. The abovementioned compound was then submitted to the General Procedure **C**, thus obtaining the title compound as a yellow solid (63% yield) after purification by silica gel column chromatography (DCM/MeOH 100:1). ^1^H NMR (300 MHz, Acetone-*d*_6_) *δ* 13.75 (s, 1H, C2′O*H*), 8.20 (d, *J* = 8.9 Hz, 1H, C6′-*H*), 8.06 (d, *J* = 15.2 Hz, 1H, CαH=Cβ*H*), 8.05 (s, 1H, C4-*H*), 7.91 (d, *J* = 15.3 Hz, 1H, Cα*H*=CβH), 7.76 (d, *J* = 8.6 Hz, 1HC6-*H*), 7.52 (d, *J* = 8.6 Hz, 1H, C7-*H*), 7.45 (d, *J* = 8.5 Hz, 2H, C2″-*H* and C6″-*H*), 7.01 (d, *J* = 8.5 Hz, 2H, C3″-*H* and C5″-*H*), 6.55 (s, 1H, C3-*H*), 6.50 (dd, *J* = 8.8, 2.2 Hz, 1H, C5′-*H*), 6.38 (d, *J* = 2.2 Hz, 1H, C3′-*H*), 3.80 (s, 3H, NC*H_3_*). ^13^C NMR (75 MHz, Acetone-*d*_6_) *δ* 191.98, 166.72, 164.56, 157.71, 146.45, 143.18, 139.77, 132.42, 130.65 (2C), 128.50, 126.72, 123.40, 122.53, 121.70, 117.04, 115.45 (2C), 113.69, 110.36, 107.72, 102.86, 101.29, 30.70. ESI-MS, *m*/*z* [*M*-H]^−^ 384.

*(E)-3-(1-Benzyl-2-(4-hydroxyphenyl)-1H-indol-5-yl)-1-(2,4-dihydroxyphenyl)prop-2-en-1-one (***28b***)*. Following the General Procedure **B**, starting from 1-(2-hydroxy-4-(methoxymethoxy)phenyl)ethan-1-one (**14a**) and 1-benzyl-2-(4-(methoxymethoxy)phenyl)-1H-indole-5-carbaldehyde (**27b**), (*E*)-3-(1-benzyl-2-(4-(methoxymethoxy)phenyl)-1H-indol-5-yl)-1-(2-hydroxy-4-(methoxymethoxy)phenyl)prop-2-en-1-one was obtained as a yellow oil (65% yield) after purification by silica gel column chromatography (Pet. Et./EtOAc 2:1). ^1^H NMR (300 MHz, Acetone-*d*_6_) *δ* 13.64 (s, 1H, C2′O*H*), 8.25 (d, *J* = 8.9 Hz, 1H, C6′-*H*), 8.13 (s, 1H, C4-*H*), 8.08 (d, *J* = 15.3 Hz, 1H, CαH=Cβ*H*), 7.92 (d, *J* = 15.3 Hz, 1H, Cα*H*=CβH), 7.78 (d, *J* = 8.8 Hz, 1H, C6-*H*), 7.46 (d, *J* = 8.6 Hz, 2H, C2″-*H* and C6″-*H*), 7.40 (d, *J* = 8.6 Hz, 1H, C7-*H*), 7.32–7.20 (m, 3H, NCH_2_*Ph*), 7.12 (d, *J* = 8.6 Hz, 2H, C3″-*H* and C5″-), 7.01 (d, *J* = 7.0 Hz, 2H, NCH_2_*Ph*), 6.70 (s, 1H, C3-*H*), 6.63 (dd, *J* = 9.0, 2.2 Hz, 1H, C5′-*H*), 6.58 (d, *J* = 2.1 Hz, 1H, C3′-*H*), 5.53 (s, 2H, NC*H_2_*Ph), 5.30 (s, 2H, OC*H_2_*OCH_3_), 5.25 (s, 2H, OC*H_2_*OCH_3_), 3.46 (s, 6H, 2xOCH_2_OC*H_3_*). ESI-MS, *m*/*z* [*M*-H]^−^ 548. The abovementioned compound was then submitted to the General Procedure **C**, thus obtaining the title compound as a yellow oil (61% yield) after purification by silica gel column chromatography (Pet. Et./EtOAc 1:1). ^1^H NMR (300 MHz, Acetone-*d*_6_) *δ* 13.71 (s, 1H, C2′O*H*), 8.17 (d, *J* = 8.9 Hz, 1H, C6′-*H*), 8.09 (s, 1H, C4-*H*), 8.04 (d, *J* = 15.3 Hz, 1H, CαH=Cβ*H*), 7.88 (d, *J* = 15.3 Hz, 1H, Cα*H*=CβH), 7.67 (d, *J* = 7.6 Hz, 1H, C6-*H*), 7.37 (m, 3H, C7-*H*, C2″-*H* and C6″), 7.32–7.20 (m, 3H, NCH_2_*Ph*), 7.01 (d, *J* = 6.9 Hz, 2H, NCH_2_*Ph*), 6.93 (d, *J* = 8.4 Hz, 2H, C3″-*H* and C5″-*H*), 6.65 (s, 1H, C3-*H*), 6.48 (dd, *J* = 8.8, 2.2 Hz, 1H, C5′-*H*),), 6.38 (d, *J* = 2.2 Hz, 1H, C3′-*H*),), 5.51 (s, 2H, NC*H_2_*Ph). ^13^C NMR (75 MHz, Acetone-*d*_6_) *δ* 192.41, 167.15, 165.02, 158.29, 146.65, 143.78, 139.73, 138.73, 132.84, 131.03, 129.32, 129.01 (2C), 127.58 (2C), 126.44 (2C), 123.77, 123.03, 122.35, 117.84, 115.94 (2C), 114.15, 111.60, 108.21, 103.33, 102.69, 102.66, 47.59. ESI-MS, *m*/*z* [*M*-H]^−^ 460

### 3.3. Antileishmanial Activity Assay

Promastigote stage of *L. infantum* (MHOM/TN/80/IPT1) were cultured in Schneider’s Drosophila medium (Lonza, Rome, Italy) supplemented with 10% heat-inactivated fetal calf serum (HyClone, Thermo Fisher Scientific, Milan, Italy) at 23 °C. The complete medium used for antileishmanial activity assay was RPMI (EuroClone SpA, Milan, Italy) supplemented with 10% heat-inactivated fetal calf serum (EuroClone), 20 mM Hepes, and 2 mM L-glutamine. To estimate the 50% inhibitory concentration (IC50), the MTT (3-[4.5-dimethylthiazol-2-yl]-2.5-diphenyltetrazolium bromide, Merk Life Science S.r.l., Milan, Italy) method was used with modifications [33]. Compounds were dissolved in DMSO and then diluted with medium to achieve the required concentrations. Drugs were placed in 96 wells round-bottom microplates, and seven serial dilutions were made. Amphotericin B was used as the reference anti-*Leishmania* drug. Parasites were diluted in complete medium to 5 × 10^6^ parasites/mL, and 100 μL of the suspension was seeded into the plates, incubated at 23 °C for 72 h, and then 20 µL of MTT solution (5 mg/mL) was added into each well for 3 h. The plates were then centrifuged, the supernatants discarded, and the resulting pellets dissolved in 100 µL of lysing buffer consisting of 20% (*w*/*v*) of a solution of SDS (Merk Life Science S.r.l.), 40% of *N*,*N*-dimethylformamide (Merk Life Science S.r.l.) in H_2_O. The absorbance was measured spectrophotometrically at a test wavelength of 550 nm and a reference wavelength of 650 nm. The results are expressed as IC_50_; each IC_50_ value is the mean ± standard deviation of separate experiments performed in duplicate.

## 4. Conclusions

We developed an optimized total synthesis of the natural chalcone lophirone E (**1**) and of analogues bearing heterocyclic **B**-rings. Our synthetic studies highlighted the utility and limitations of the TFP *O*-protecting group for the preparation of chalcone derivatives and allowed the synthesis of a set of analogues for early SAR investigation. The antileishmanial activity of the compounds is promising and poses the basis for the design of novel analogues endowed with higher potency worthy of further biological investigation and the optimization of selected drug-like properties, including an evaluation of the toxicological profile. To the best of our knowledge, this is the first report of the synthesis of chalcones bearing a 2-arylindol-5-yl-moiety, and their interesting antileishmanial activity makes this class of chalcones worthy of further investigation.

## Figures and Tables

**Table 1:**
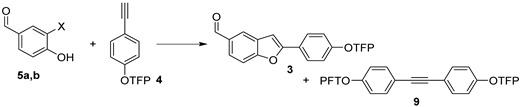
Investigation of the reaction conditions of the one-pot Sonogashira coupling–intramolecular cyclization reaction.

Entry	X	Catalyst	Solvent	Time (h)	Yield 3 (%)	Yield 9 (%)
1	Br	Pd(PPh_3_)_4_	Et_3_N	3	n.d.	n.d. *
2	Br	Pd(PPh_3_)_4_	Et_3_N	24	n.d.	74
3	Br	Pd(dppf)Cl_2_	Degassed Et_3_N	12	traces **	41
4	Br	Cl_2_Pd(PPh_3_)_2_	THF/degassed Et_3_N	12	n.d.	63
5	Br	Pd(PPh_3_)_4_	Degassed Et_3_N	3	8	n.d.
6	Br	Pd(PPh_3_)_4_	Degassed Et_3_N	12	12	15
7	Br	Pd(PPh_3_)_4_	Degassed Et_3_N	20	39	40
8	I	Pd(PPh_3_)_4_	Degassed Et_3_N	12	83	n.d.

* n.d. = not detectable; ** traces = yield < 5%.

**Table 2:** Activity of compounds **1**, **16**–**18**, **21a**,**b**, **25a**,**b**, **28a**,**b**, **29** and of reference compound amphotericin B against *L. infantum* promastigotes.

Cpd	Structure	*L. infantum* * Promastigotes IC_50_ (µM)
**1**	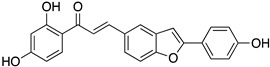	15.36 ± 6.55
**16**	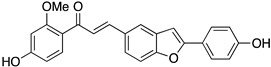	>65
**17**	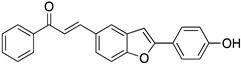	>65
**18**	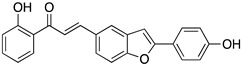	13.92 ± 2.55
**21a**	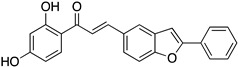	15.55 ± 7.72
**21b**	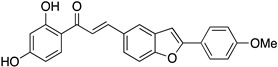	>65
**25a**	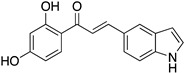	56.57 ± 5.69
**25b**	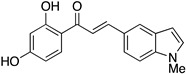	>65
**28a**	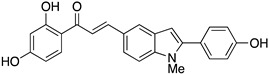	27.19 ± 5.79
**28b**	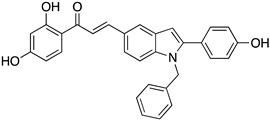	15.93 ± 2.10
29 **	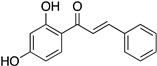	47.62 ± 4.00

* Data are the mean and SD of three experiments; Amphotericin B IC_50_ = 0.13 ± 0.02 µg/mL ** Prepared as described in the Supporting Material.

## Data Availability

Not applicable.

## Supplementary Materials

The following supporting information can be downloaded online: Scheme S1 (Synthesis of compound **29**); Figures S1–S21 (^1^H and ^13^C NMR spectra of compounds submitted to biological investigation).

## Sample Availability

Samples of the compounds are not available.
