# Supplementary material for: Total Synthesis of the Natural Chalcone Lophirone E, Synthetic Studies toward Benzofuran and Indole-Based Analogues, and Investigation of Anti-Leishmanial Activity
Source: Molecules. 2022 Jan 11;27(2):463. doi: 10.3390/molecules27020463 (PMC8778746; doi:10.3390/molecules27020463)
Supplement: Supplementary file 1 [file molecules-27-00463-s001.zip › molecules-1527742-supplementary.pdf]

molecules

MDPI

Article

Supplementary Materials

Total Synthesis of the Natural Chalcone Lophirone E and Synthetic Studies toward Benzofuran and Indole-based Analogues: Investigation of their anti-Leishmanial Activity

Luca Pozzetti,[1] Roberta Ibba,[1] Sara Rossi,[1] Orazio Taglialatela-Scafati,[2] Donatella Taramelli,[3] Nicoletta Basilico,[4] Sarah D'Alessandro[3], Silvia Parapini [5], Stefania Butini,[1] Giuseppe Campiani,[1*] Sandra Gemma,[1*]

[1] Department of Biotechnology, Chemistry and Pharmacy, University of Siena, Via Aldo Moro 2, 53100 Siena, Italy; luca.pozzetti@student.unisi.it (L.P.); roberta.ibba@unisi.it (R.I.); rossi115@student.unisi.it (R.S.); butini3@unisi.it (S.B.); campiani@unisi.it (G.C.); gemma@unisi.it (G.S.)
[2] Department of Pharmacy, School of Medicine and Surgery, University of Naples Federico II, Via D. Montesano 49, 80131 Naples, Italy; scatagli@unina.it (O.T.-S.)
[3] Department of Pharmacological and Molecular Sciences, University of Milan, Via Pascal 36, 20133 Milano, Italy donatella.taramelli@unimi.it, sarah.dalessandro@unimi.it (S.D.)
[4] Department of Biomedical, Surgical and Dental Sciences, University of Milan, Via Pascal 36, 20133 Milano, Italy nicoletta.basilico@unimi.it (N.B.)
[5] Department of Biomedical Sciences for Health, University of Milan, Via Mangiagalli 31, 20133 Milano silvia.parapini@unimi.it (S.P.)
* Correspondence: gemma@unisi.it; campiani@unisi.it

1. Chemistry

1.1. Synthesis of reference chalcone **29**

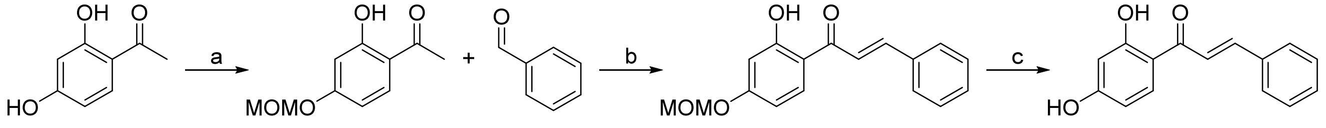

Scheme S1. *Reagents and Conditions*: **a**) MOMCl, DIPEA, dry DCM, 25 °C, 2 h; **b**) NaOH (5 M), EtOH, 0 °C to 25 °C, 72 h; **c**) HCl (12 M), MeOH/THF, 0 °C to 40 °C, 1 h.

1.1.1. Experimental Procedures

*1-(2-Hydroxy-4-(methoxymethoxy)phenyl)ethan-1-one (**14a**).* To a solution of 1-(2,4-dihydroxyphenyl)ethan-1-one (**10**, 1 eq., 1 mmol) in anhydrous DCM (10 mL), DIPEA (1.5 eq., 1.5 mmol) was added, followed by chloromethyl methyl ether (1.5 eq., 1.5 mmol). The mixture was stirred at 25 °C for 2 h. Reaction mixture was washed with HCl (1 M) (2x5 mL) and then with $NaHCO_3$ (s.s., 5 mL), and brine (5 mL). The organic phase was dried over Na_2SO_4 and evaporated under reduced pressure to obtain the title compound with no further purification (quantitative yield) as a yellow oil. ^{1}H NMR (300 MHz, $CDCl_3$) *δ* 12.60 (s, 1H, C2′O*H*), 7.65 (d, *J* = 8.8 Hz, 1H, C6′-*H*), 6.59 (d, *J* = 2.4 Hz, 1H, C3′-*H*), 6.55 (dd, *J* = 8.8, 2.4 Hz, 1H, C5′-*H*), 5.20 (s, 2H, OC*H*$_2$OCH$_3$), 3.47 (s, 3H, OCH$_2$OC*H*$_3$), 2.56 (s, 3H, COC*H*$_3$). ESI-MS, *m*/*z* [*M*-H]$^-$ 195.

*(E)-1-(2-hydroxy-4-(methoxymethoxy)phenyl)-3-phenylprop-2-en-1-one (**31**).* To a solution of 1-(2-Hydroxy-4-(methoxymethoxy)phenyl)ethan-1-one (**14a**) (1 eq., 1 mmol) in absolute EtOH (3 mL), cooled to 0 °C, $NaOH_{aq.}$ (5 M, 5 mmol) was added dropwise. The mixture was stirred at 0 °C for 30 minutes and then a solution of benzaldehyde (**30**) (1.2 eq., 1.2 mmol) in absolute EtOH (1.5 mL) was added dropwise. The ice bath was removed, and the mixture was stirred at 25 °C for 48 h. The reaction was acidified with HCl (1 M) and extracted with EtOAc (3x5 mL). The combined organic phases were washed with $NaHCO_3$ (s.s., 10 mL) and brine (5 mL), dried over Na_2SO_4 and evaporated under reduced pressure. The residue was purified by silica gel column chromatography (Pet. Et./EtOAc 2:1) to afford the title compound as a yellow solid (85% yield). ^{1}H NMR (300 MHz, $CDCl_3$) δ 13.27 (s, 1H, C2′O*H*), 7.89 (d, *J* = 15.7 Hz, 1H, CαH=Cβ*H*), 7.83 (d, *J* = 9.0 Hz, 1H, C6′-*H*), 7.69 – 7.62 (m, 2H, Ar-*H*), 7.58 (d, *J* = 15.5 Hz, 1H, Cα*H*=CβH), 7.47 – 7.35 (m, 3H, Ar-*H*), 6.65 (d, *J* = 2.3 Hz, 1H, C3′-*H*), 6.60 (dd, *J* = 9.0, 2.2 Hz, 1H, C5′-*H*), 5.22 (s, 2H, OC*H*$_2$OCH$_3$), 3.49 (s, 3H, OCH$_2$OC*H*$_3$). ESI-MS, *m*/*z* [*M*-H]$^-$ 283.

*(E)-1-(2,4-dihydroxyphenyl)-3-phenylprop-2-en-1-one (**29**,* CAS registry number 25515-43-9*).* To a 0 °C cooled solution of (E)-1-(2-hydroxy-4-(methoxymethoxy)phenyl)-3-phenylprop-2-en-1-one (**31**) (1 eq., 1 mmol) in MeOH/THF (1:1, 20 mL in total), HCl (12 M, 20 eq., 20 mmol) was added dropwise. The ice bath was removed, and the reaction mixture was heated to 40 °C for 3 h. After cooling, $NaHCO_3$ (s.s.) was carefully added until pH=7 and the aqueous phase was extracted with EtOAc (3x10 mL). The combined organic phases were washed with brine (15 mL), dried over Na_2SO_4 and evaporated under reduced pressure. The residue was purified by silica gel column chromatography (DCM/MeOH 100:1) to afford the title compound as a yellow solid (quantitative yield). ^{1}H NMR (300 MHz, $CDCl_3$) δ 13.37 (s, 1H, C2′O*H*), 7.89 (d, *J* = 15.9 Hz, 1H, CαH=Cβ*H*), 7.83 (d, *J* = 8.9 Hz, 1H, C6′-*H*), 7.74 – 7.61 (m, 2H, Ar-*H*), 7.57 (d, *J* = 15.5 Hz, 1H, Cα*H*=CβH), 7.51 – 7.35 (m, 3H, Ar-*H*), 6.45 (m, 2H). ^{13}C NMR (75 MHz, $CDCl_3$) δ 191.99, 166.40, 162.70, 144.67, 134.70, 132.02, 130.72, 128.99 (2C), 128.54 (2C), 120.22, 114.49, 107.80, 103.81. ESI-MS, *m*/*z* [*M*-H]$^-$ 239.

1.2. Spectral data of final compounds

1.2.1. **Compound 1**

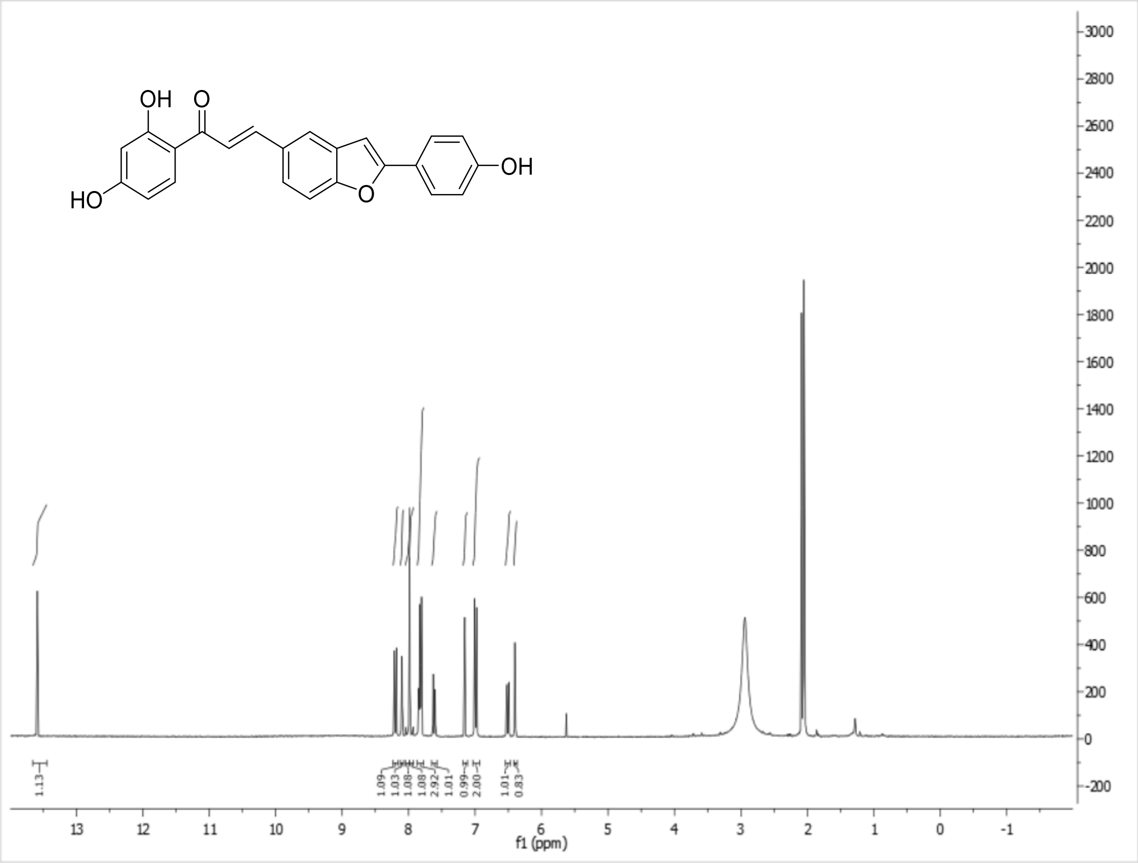

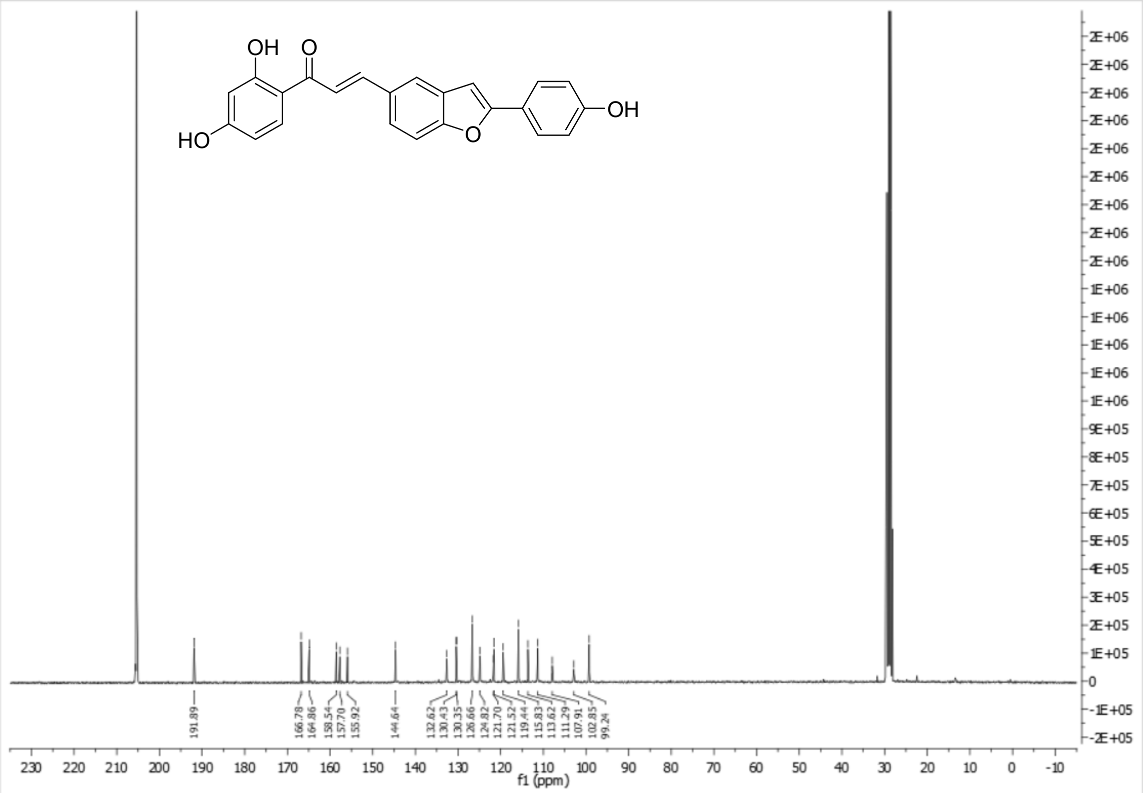

Figure S1. ^{1}H NMR (300 MHz, Acetone-d_6) spectrum of lophirone E (**1**).

Figure S2. ^{13}C NMR (75 MHz, Acetone-*d*6) spectrum of lophirone E (**1**).

1.2.2. **Compound 16**

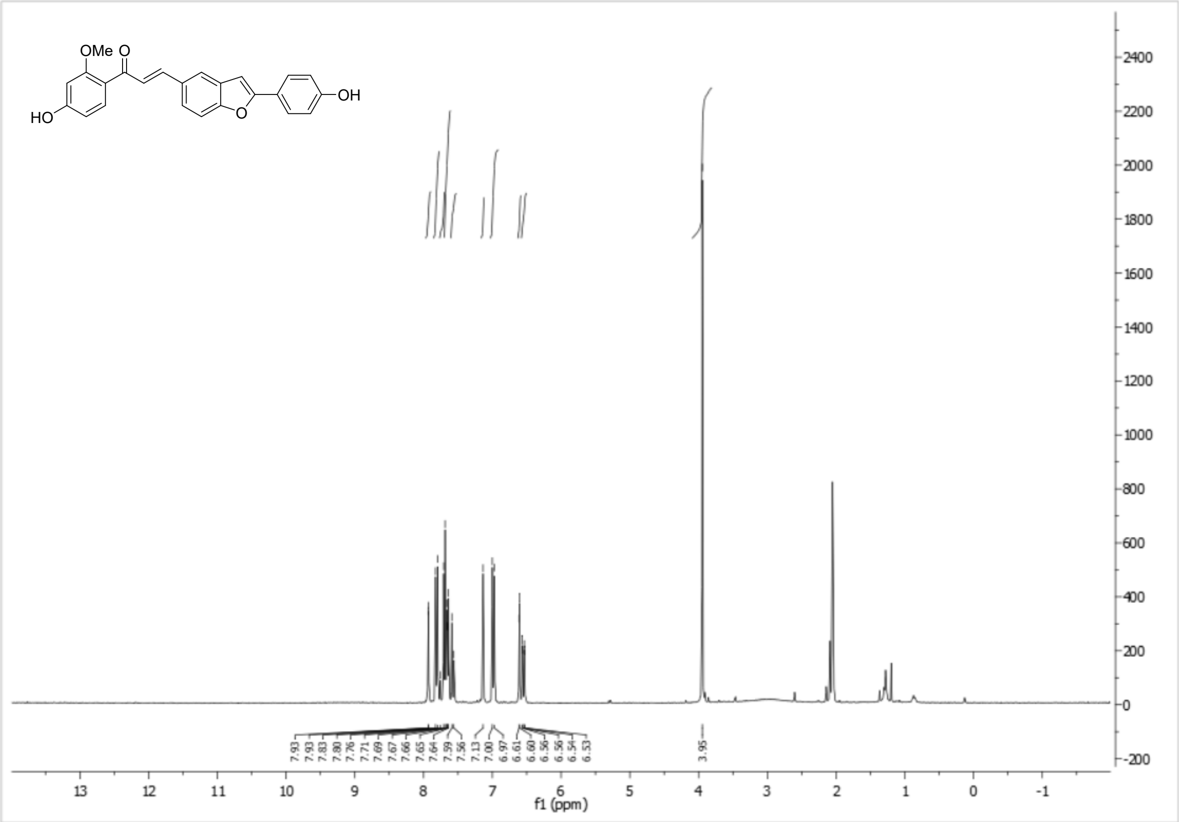

Figure S3. ^{1}H NMR (300 MHz, Acetone-*d*6) spectrum of compound **16**.

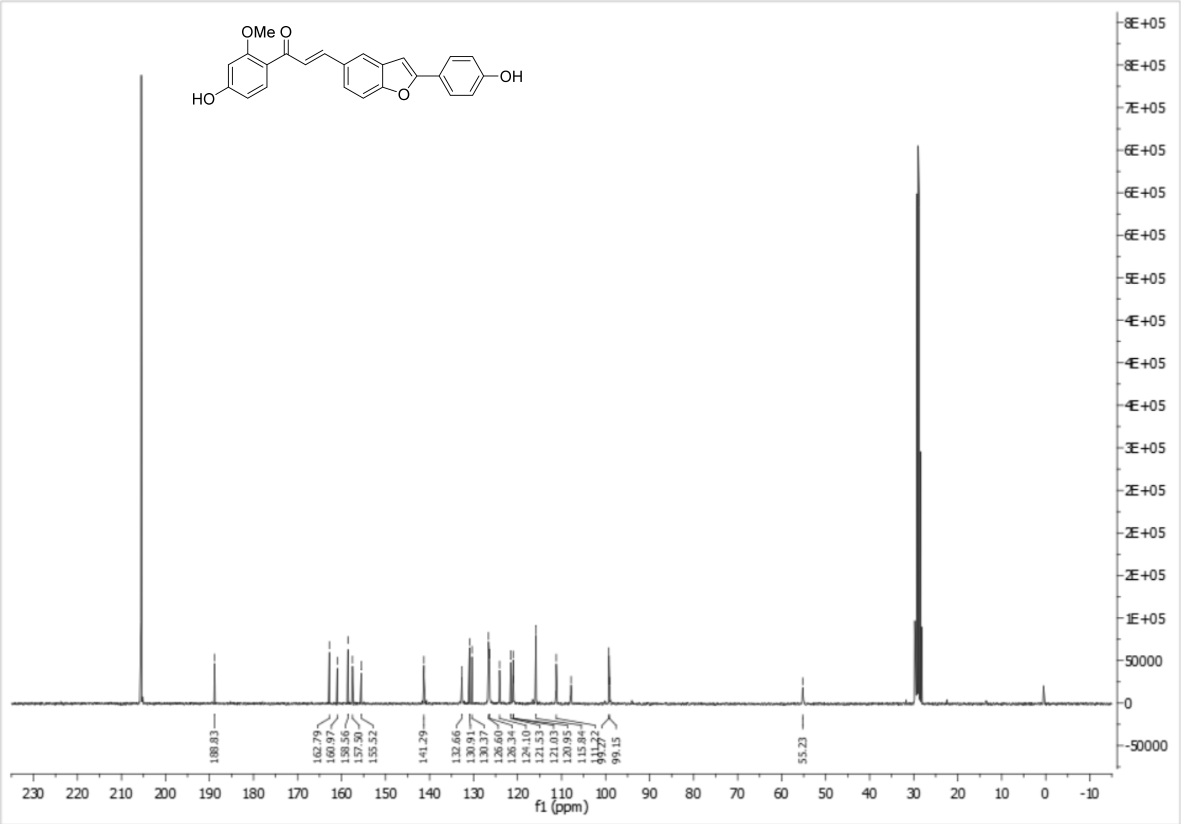

Figure S4. ^{13}C NMR (75 MHz, Acetone-*d*6) spectrum of compound **16**.

1.2.3. **Compound 17**

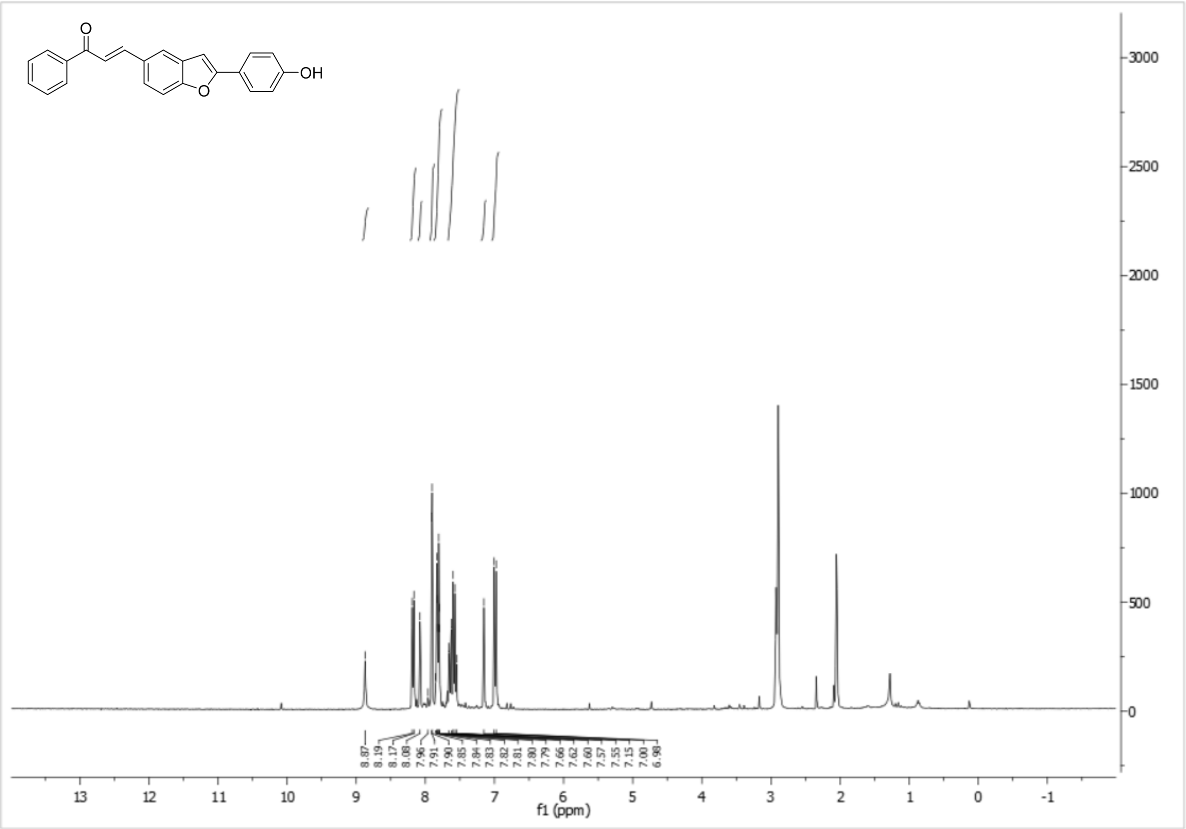

Figure S5. ^{1}H NMR (300 MHz, Acetone-*d*$_6$) spectrum of compound **17**.

1.2.4. **Compound 18**

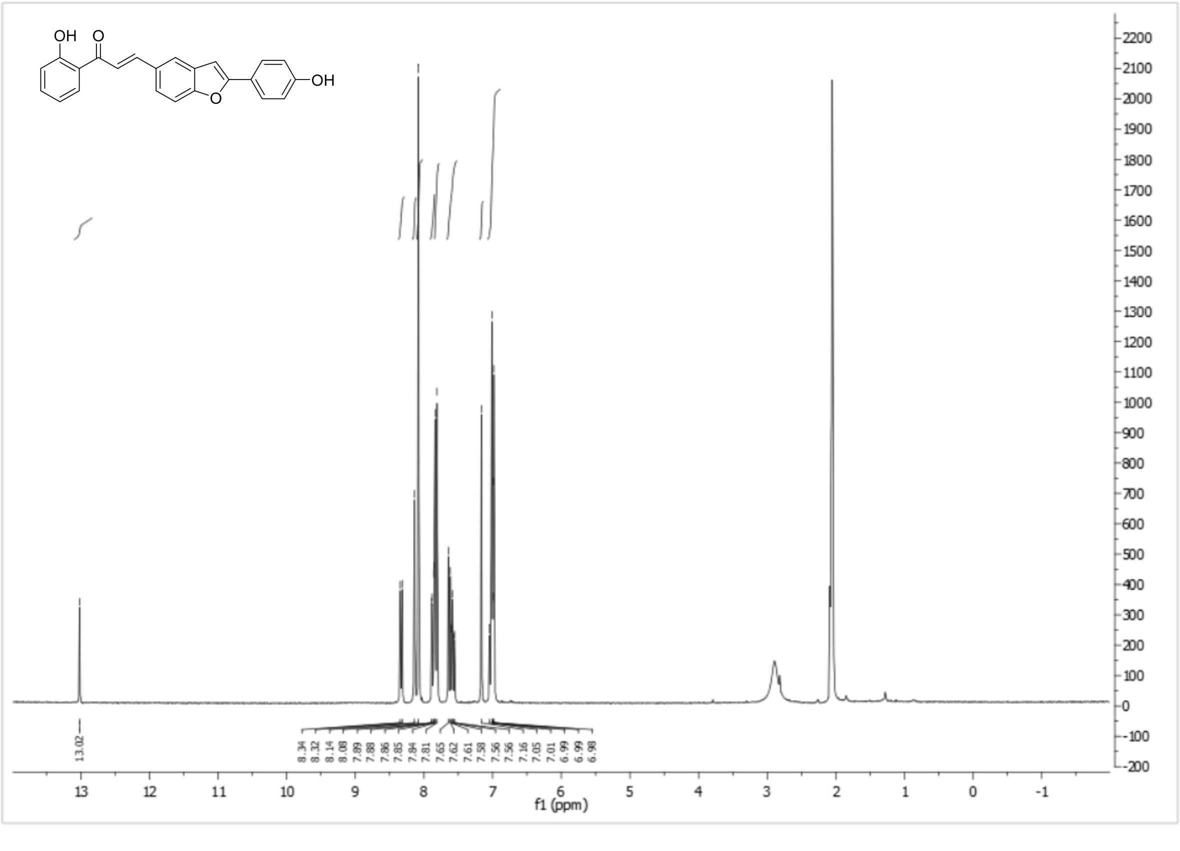

Figure S6. ¹H NMR (300 MHz, Acetone-*d*6) spectrum of compound **18**.

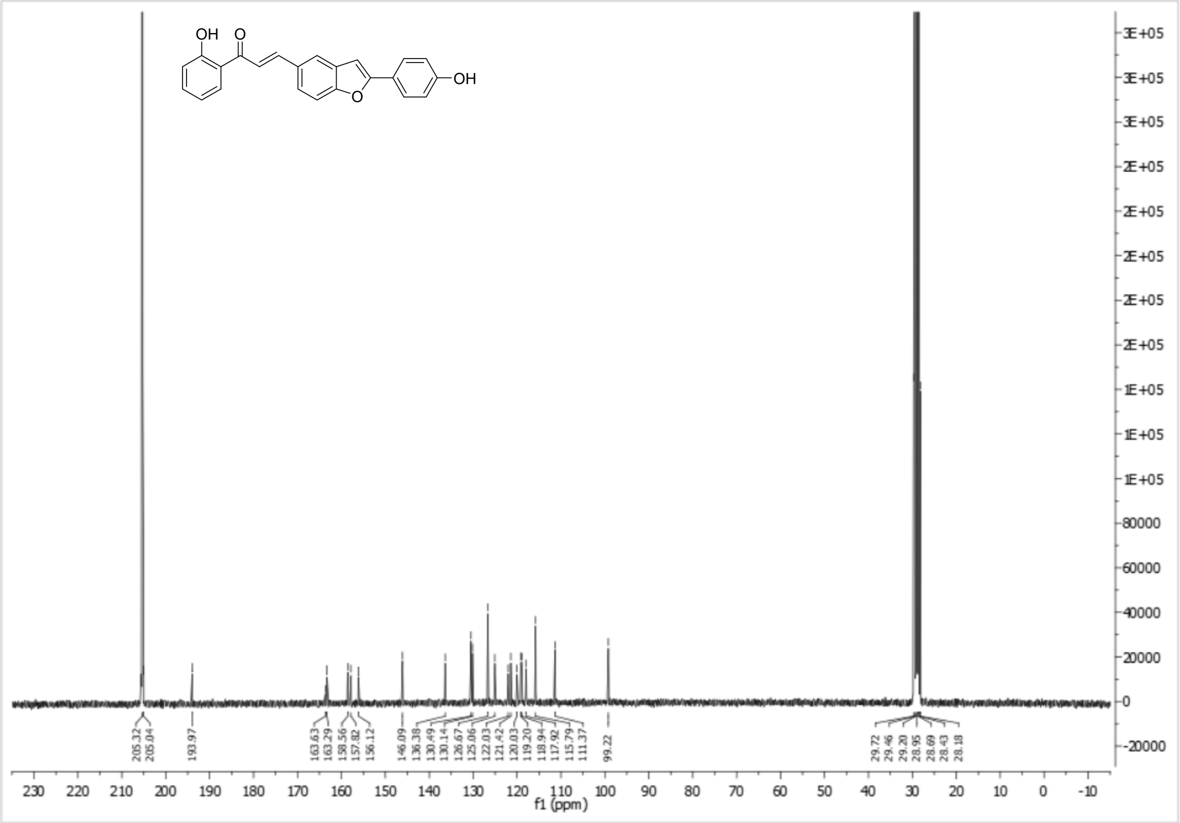

Figure S7. ¹³C NMR (75 MHz, Acetone-*d*6) spectrum of compound **18**.

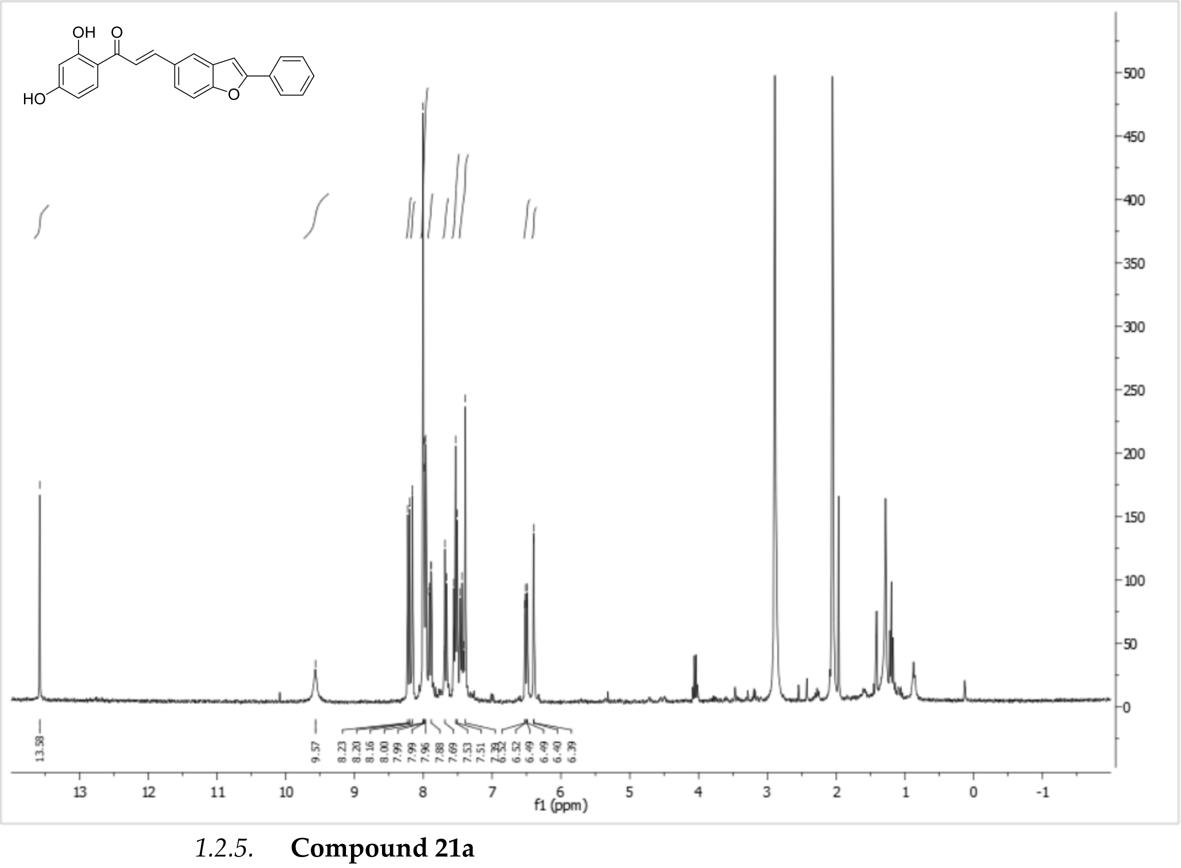

1.2.5. **Compound 21a**

Figure S8. ¹H NMR (300 MHz, Acetone-*d*6) spectrum of compound **21a**.

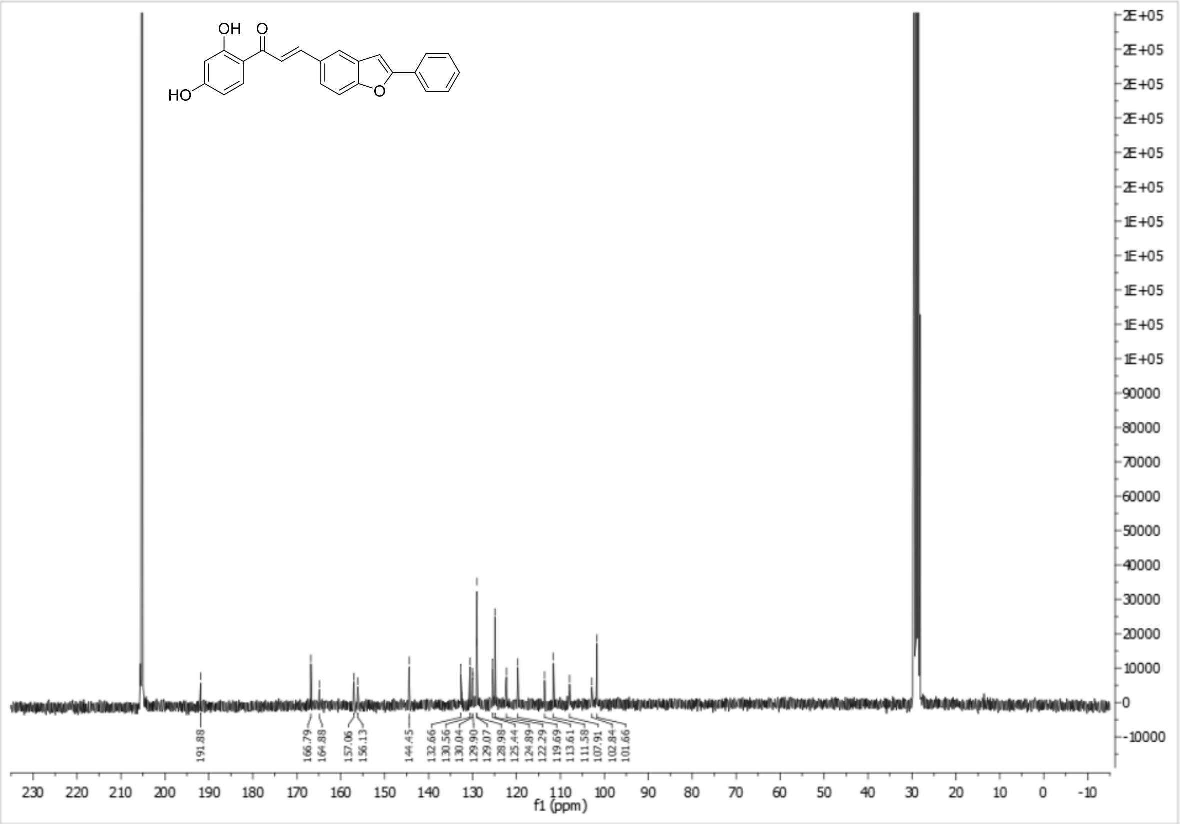

Figure S9. ^{13}C NMR (75 MHz, Acetone-d_6) spectrum of compound **21a**.

1.2.6. **Compound 21b**

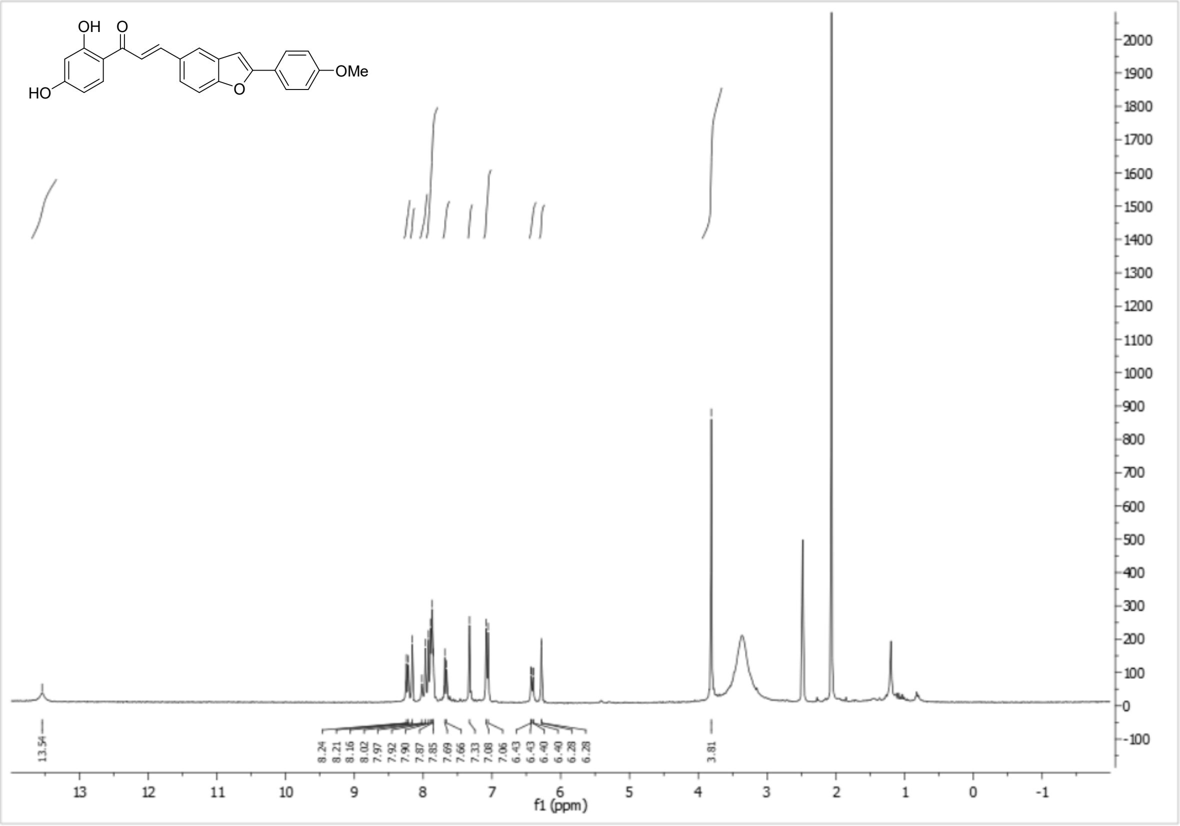

Figure S10. ^{1}H NMR (300 MHz, DMSO-d_6) spectrum of compound **21b**.

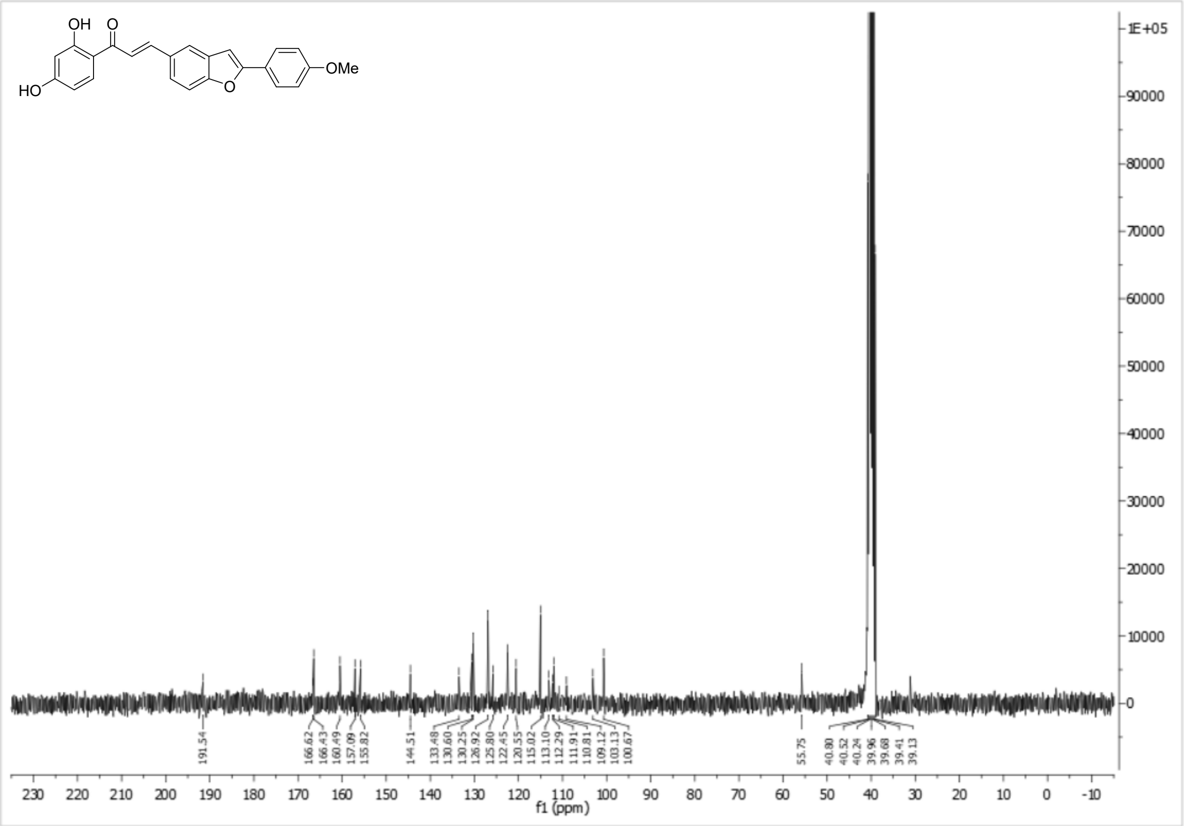

Figure S11. ^{13}C NMR (75 MHz, Acetone-d_6) spectrum of compound **21b**.

1.2.7. Compound 25a

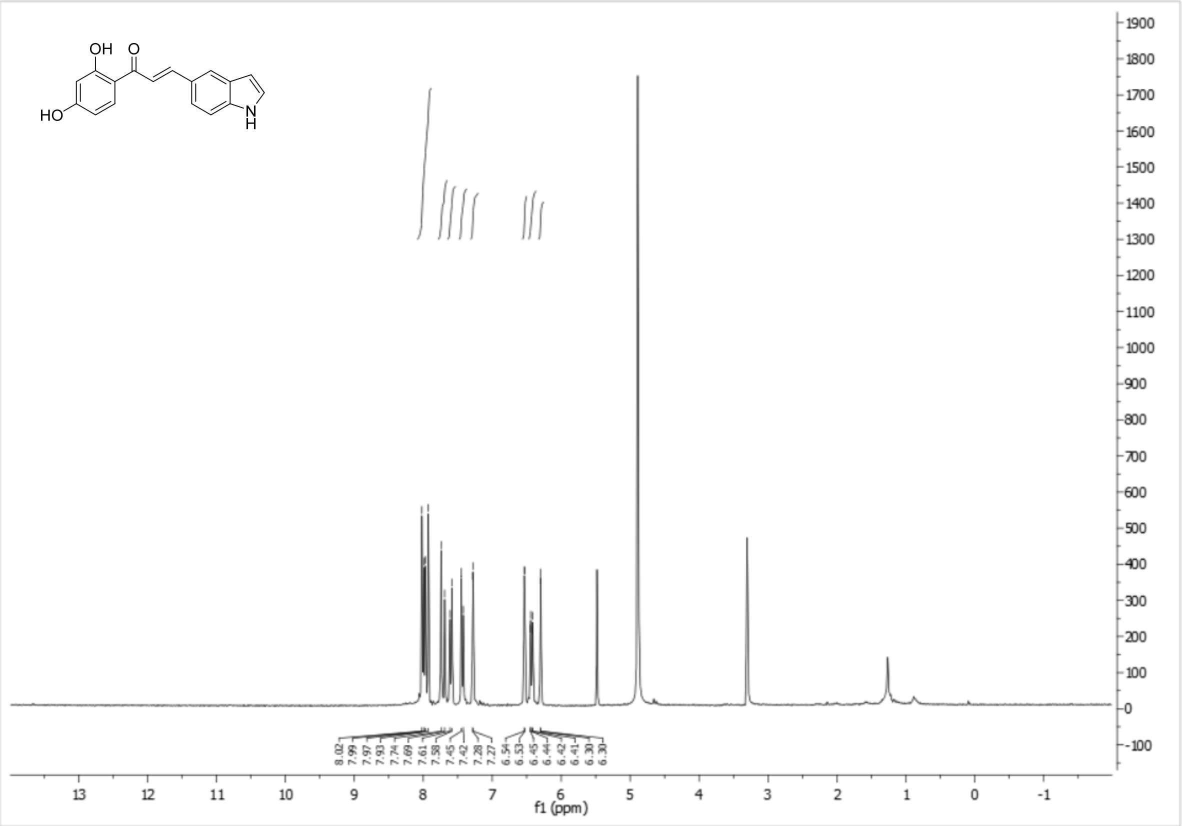

Figure S12. ^{1}H NMR (300 MHz, CD_3OD) spectrum of compound **25a**.

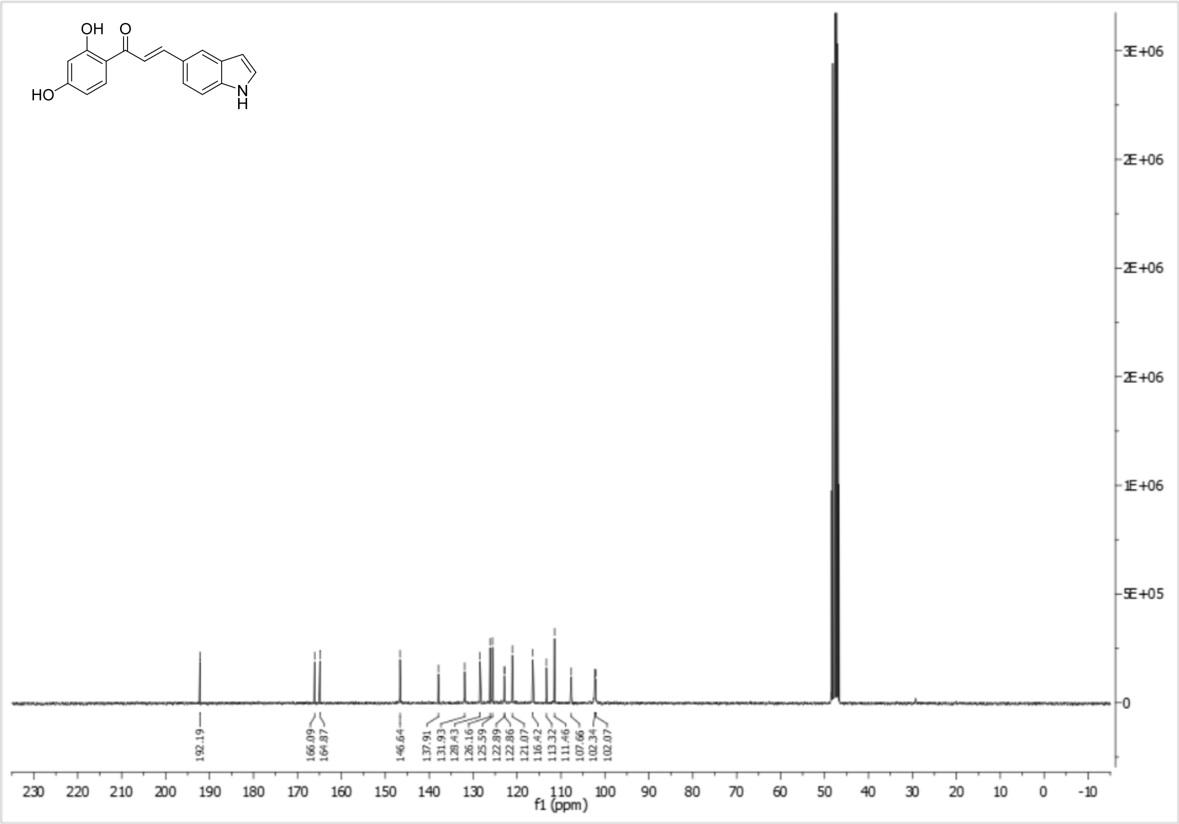

Figure S13. ^{13}C NMR (75 MHz, CD_3OD) spectrum of compound **25a**.

1.2.8. **Compound 25b**

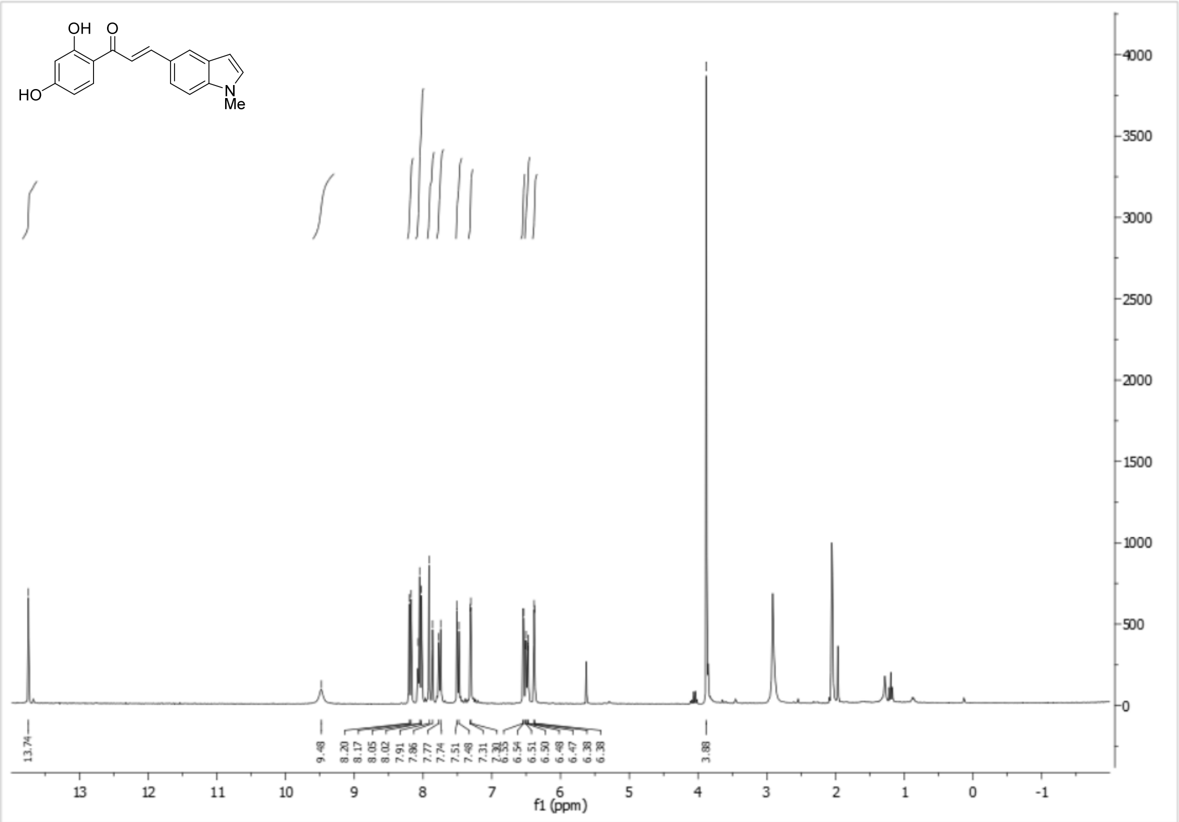

Figure S14. ^{1}H NMR (300 MHz, Acetone-d_6) spectrum of compound **25b**.

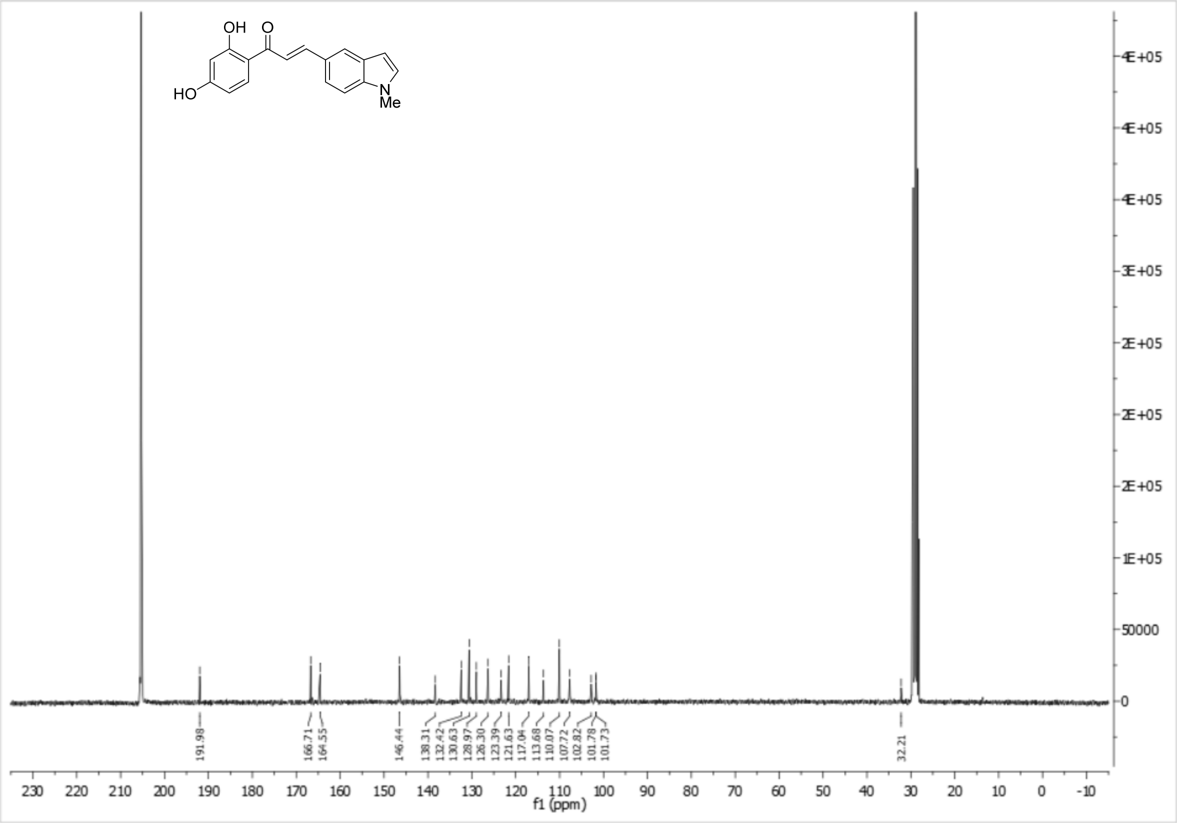

Figure S15. ^{13}C NMR (75 MHz, Acetone-d_6) spectrum of compound **25b**.

1.2.9. **Compound 28a**

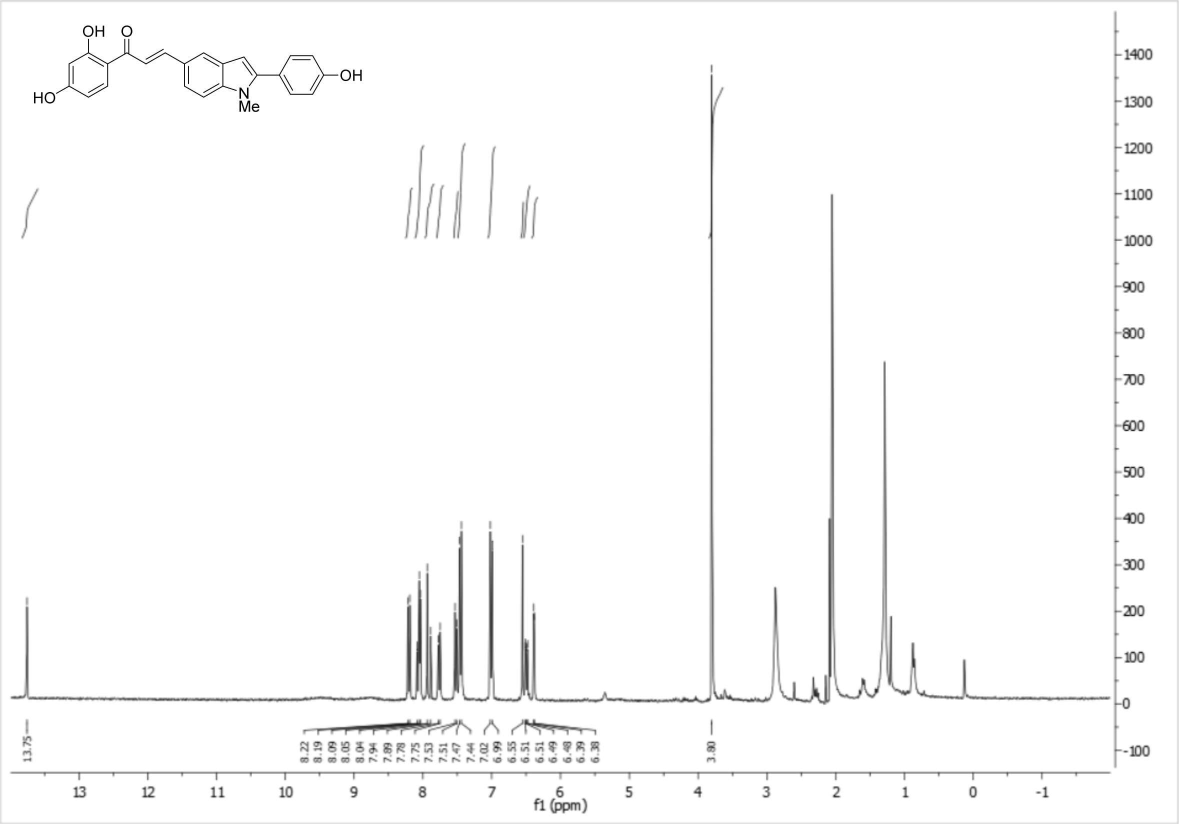

Figure S16. ^{1}H NMR (300 MHz, Acetone-d_6) spectrum of compound **28a**.

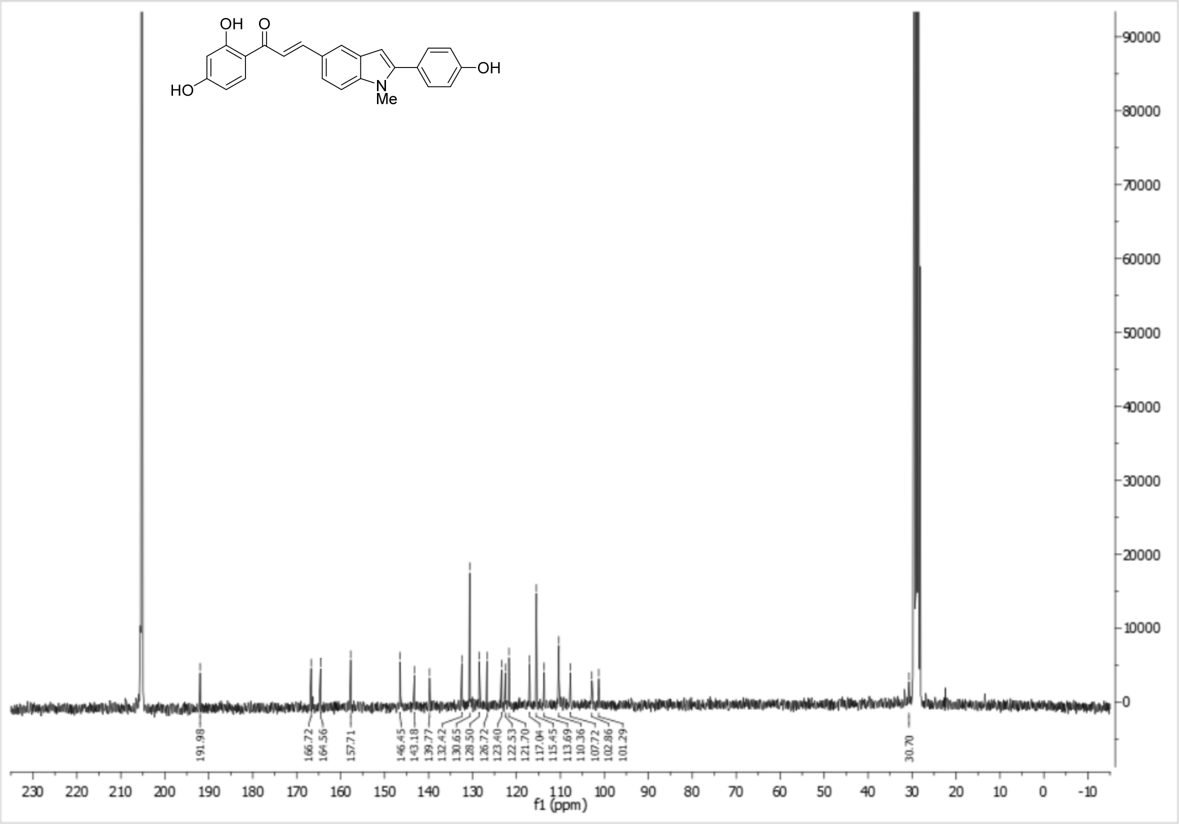

Figure S17. ^{13}C NMR (75 MHz, Acetone-d_6) spectrum of compound **28a**.

1.2.10. **Compound 28b**

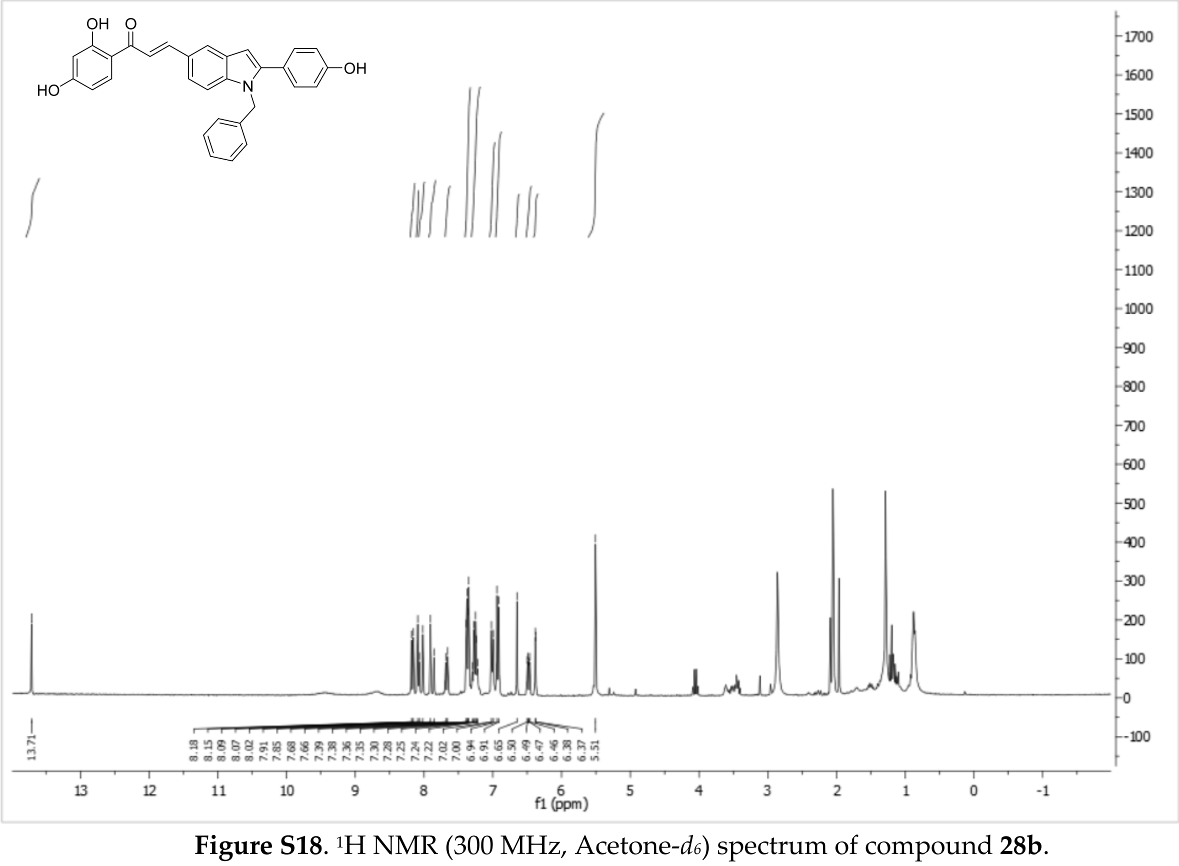

Figure S18. ^{1}H NMR (300 MHz, Acetone-d_6) spectrum of compound **28b**.

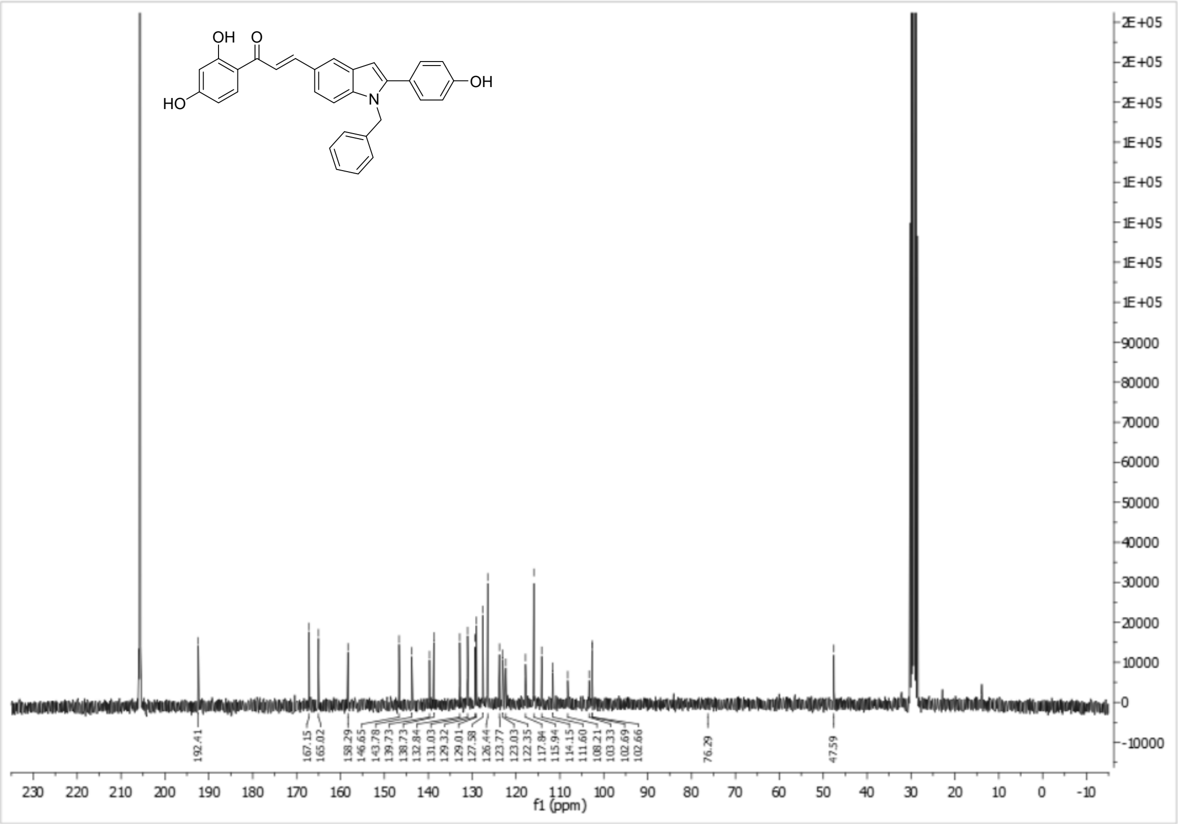

Figure S19. ^{13}C NMR (75 MHz, Acetone-d_6) spectrum of compound **28b**.

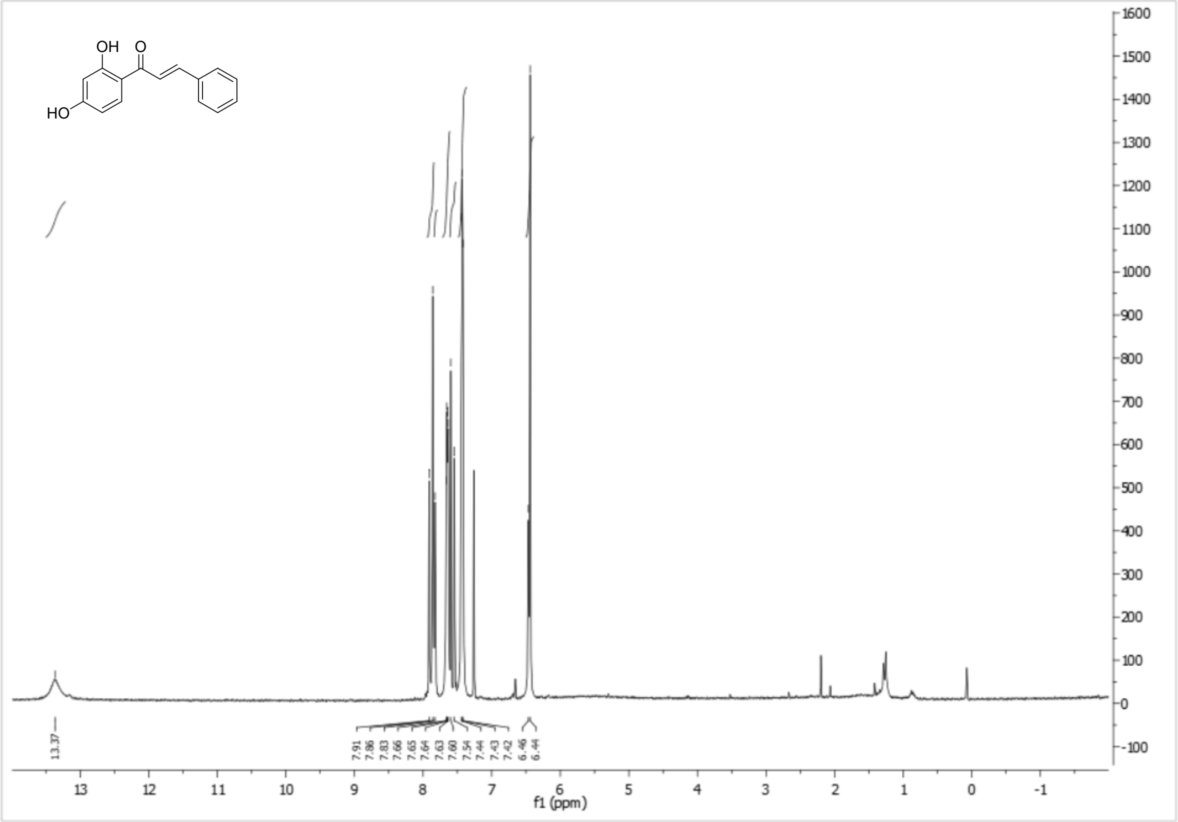

1.2.11. **Reference compound 29**

Figure S20. ^{1}H NMR (300 MHz, $CDCl_3$) spectrum of reference compound **29**.

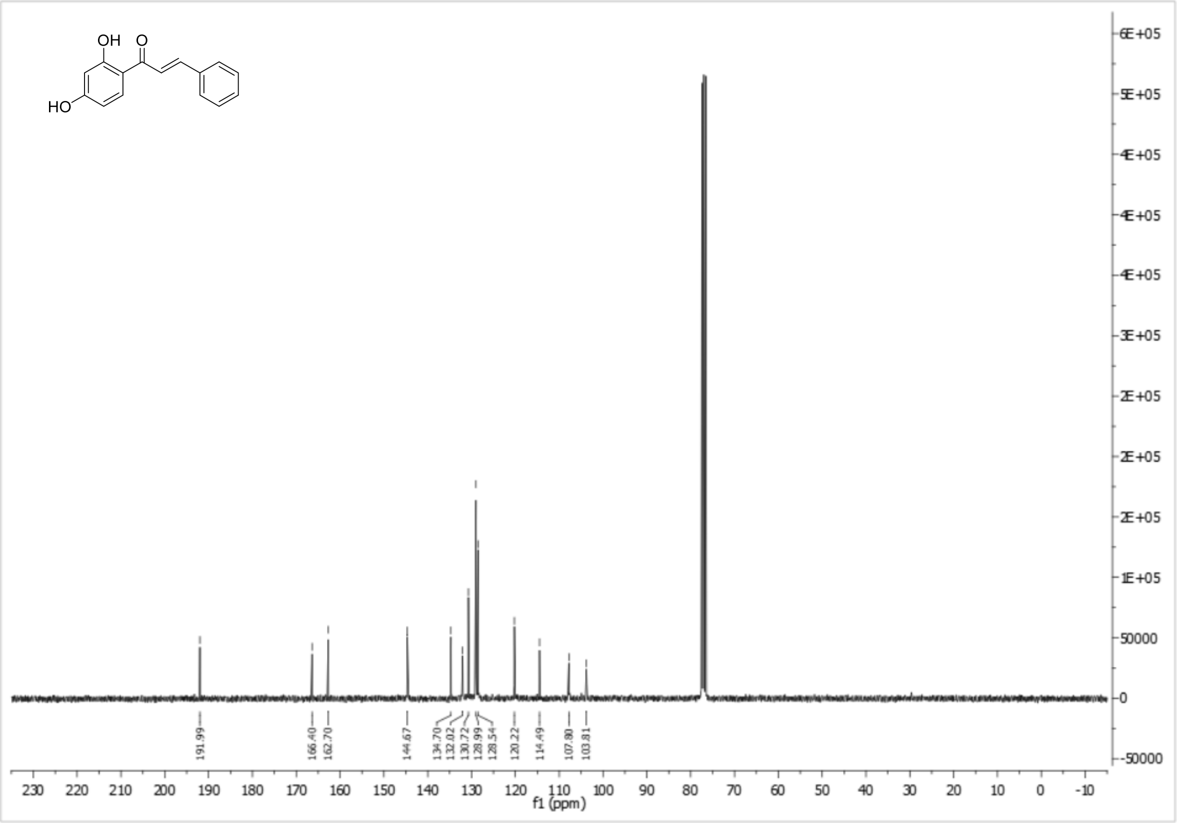

Figure S21. ^{13}C NMR (75 MHz, $CDCl_3$) spectrum of reference compound **29**.
